# Influence of Inflammation on Cytochromes P450 Activity in Adults: A Systematic Review of the Literature

**DOI:** 10.3389/fphar.2021.733935

**Published:** 2021-11-16

**Authors:** Camille Lenoir, Victoria Rollason, Jules A. Desmeules, Caroline F. Samer

**Affiliations:** ^1^ Division of Clinical Pharmacology and Toxicology, Department of Anesthesiology, Pharmacology, Intensive Care, and Emergency Medicine, Geneva University Hospitals, Geneva, Switzerland; ^2^ Institute of Pharmaceutical Sciences of Western Switzerland (ISPSO), School of Pharmaceutical Sciences, University of Geneva, Geneva, Switzerland; ^3^ Faculty of Medicine, University of Geneva, Geneva, Switzerland

**Keywords:** inflammation, cytochrome P450, pharmacokinetic, disease-drug interaction, cytokines

## Abstract

**Background:** Available in-vitro and animal studies indicate that inflammation impacts cytochromes P450 (CYP) activity *via* multiple and complex transcriptional and post-transcriptional mechanisms, depending on the specific CYP isoforms and the nature of inflammation mediators. It is essential to review the current published data on the impact of inflammation on CYP activities in adults to support drug individualization based on comorbidities and diseases in clinical practice.

**Methods:** This systematic review was conducted in PubMed through 7th January 2021 looking for articles that investigated the consequences of inflammation on CYP activities in adults. Information on the source of inflammation, victim drugs (and CYPs involved), effect of disease-drug interaction, number of subjects, and study design were extracted.

**Results:** The search strategy identified 218 studies and case reports that met our inclusion criteria. These articles were divided into fourteen different sources of inflammation (such as infection, autoimmune diseases, cancer, therapies with immunomodulator…). The impact of inflammation on CYP activities appeared to be isoform-specific and dependent on the nature and severity of the underlying disease causing the inflammation. Some of these drug-disease interactions had a significant influence on drug pharmacokinetic parameters and on clinical management. For example, clozapine levels doubled with signs of toxicity during infections and the concentration ratio between clopidogrel’s active metabolite and clopidogrel is 48-fold lower in critically ill patients. Infection and CYP3A were the most cited perpetrator of inflammation and the most studied CYP, respectively. Moreover, some data suggest that resolution of inflammation results in a return to baseline CYP activities.

**Conclusion:** Convincing evidence shows that inflammation is a major factor to be taken into account in drug development and in clinical practice to avoid any efficacy or safety issues because inflammation modulates CYP activities and thus drug pharmacokinetics. The impact is different depending on the CYP isoform and the inflammatory disease considered. Moreover, resolution of inflammation appears to result in a normalization of CYP activity. However, some results are still equivocal and further investigations are thus needed.

## Introduction

Cytochromes P450 (CYP) are the major drug-metabolizing enzymes (DME) responsible for 75% of drug metabolism, making them decisive in the efficacy and safety of drugs (Wienkers and Heath, 2005). The interindividual variability in CYP activity is influenced by genetic factors, environmental factors and comorbidities (Lynch and Price, 2007). CYP genetic polymorphisms are well described, resulting in major functional differences (Zhou et al., 2017). CYP are also impacted by drug-drug interactions (DDIs) and several widely used drugs were removed from the market because of serious adverse drug reactions (ADRs) due to DDIs via the CYPs (Wilkinson, 2005). Therefore, the Food and Drug Administration (FDA) requires *in-vitro* evaluation of potential DDIs during the course of drug development (Kato, 2020; Food and Drug Administration).

A less well described but increasingly studied source of modulation of CYP activity and recently reviewed is that of endogenous inflammatory markers (de Jong et al., 2020; Stanke-Labesque et al., 2020). Inflammation is a response to endogenous or exogenous aggression that can be acute or chronic. It is prominent in many diseases, such as infection, trauma, surgery, arthritis, asthma, atherosclerosis, autoimmune disease, various immunologically mediated and crystal-induced inflammatory conditions, diabetes and cancer, to name a few (Gabay and Kushner, 1999; Germolec et al., 2018; Stavropoulou et al., 2018). This universal protective response involves innate and adaptative immunity and is present in virtually all tissues. Acute changes can be associated with variation in the concentrations of several plasma proteins, the acute-phase proteins (APP), and numerous behavioral, physiological, biochemical and nutritional changes (Gabay and Kushner, 1999). Cytokines are the main stimulators of APP production, and interleukin-6 (IL-6) is the key stimulator of APP while other cytokines (IL-1β, Tumor Necrosis Factor α, interferon-γ, transforming growth factor β and possible IL-8) influence APP subgroups (Gabay and Kushner, 1999). Thus, inflammation is a complex and well-orchestrated process involving many cell types and molecules that function as a cascade network, some of which initiate, amplify or sustain the process and others attenuate or resolve it (Gabay and Kushner, 1999; Stanke-Labesque et al., 2020).

Inflammation can impact drug PK through multiple mechanisms which typically occur in the liver, kidney, or intestinal epithelial cells (Stavropoulou et al., 2018; de Jong et al., 2020; Stanke-Labesque et al., 2020). The metabolic activities of CYPs are suppressed by inflammation in most cases, but some CYPs may be induced or remain unaffected (Morgan, 2001; de Jong et al., 2020; Stanke-Labesque et al., 2020). The positive and negative control of gene transcription is generally achieved by the interaction of regulatory proteins with specific DNA sequences on the regulated genes (Morgan, 1997). The impact of inflammation on the metabolic activity of CYPs has been studied in various *in-vitro* and animal models of inflammation, including trauma, infection and administration of endotoxin or cytokines (de Jong et al., 2020; Stanke-Labesque et al., 2020). Information available in the literature suggests that this impact on PK is triggered by cytokines and their intracellular signaling, directly or *via* interaction with the nuclear receptor pathway, on drug transporters and metabolizing enzymes (Liptrott and Owen, 2011; de Jong et al., 2020; Stanke-Labesque et al., 2020). Importantly, no single common pathway has been identified to explain the changes in the entire CYP family and involves different mediators but also different transcription factors (Renton, 2005; de Jong et al., 2020; Stanke-Labesque et al., 2020). Different effects of cytokines are observed in different cell types, which could be explained by a difference in the way intracellular signals from cytokine receptors are generated (Liptrott and Owen, 2011). Different cytokines exhibit a widely different spectrum of activity trough individual CYP isoforms and many different transcription factors (Morgan, 1997; Ruminy et al., 2001; Renton, 2005; Liptrott and Owen, 2011). Their activation by cytokines have been implicated in the downregulation and transcriptional regulation of different CYP isoforms (Morgan, 1997; Ruminy et al., 2001; Renton, 2005; Liptrott and Owen, 2011). Regulation of CYP during inflammation can occur trough pre- and post-transcriptional mechanisms that are cytokine and CYP specific (de Jong et al., 2020; Stanke-Labesque et al., 2020). Pre-transcriptional mechanisms currently described in the literature include transcriptional downregulation of transcription factors, interference with dimerization/translocation of (nuclear) transcription factors, altered liver-enriched C/EBP signaling, and direct regulation by NF-κB (de Jong et al., 2020). Overall, three main mechanisms have been described to explain the downregulation of inflammation in drug metabolizing enzyme and transporters expression and activity, namely inhibition of drug metabolizing enzyme transcription, epigenetic modifications in genes as a result of DNA methylation, modification of histone patterns, release of microRNA and NO-dependent proteasome degradation, which is a post-transcriptional mechanism (Stanke-Labesque et al., 2020).

Therefore, the aim of this systemic review is to evaluate the impact of inflammation on CYP activity in the adult population.

## Methods

The method used to manage the literature search was based on the Preferred Reporting Items for Systematic Review and Meta-Analyses (PRISMA) statement (Moher et al., 2009). The detailed PICOS framework (i.e., participants, interventions, comparisons, outcomes, study design) was used as follows: Participants: adults with source of inflammation, -Intervention: victim drugs and CYPs concerned, -Comparison: healthy adults or before the onset of inflammation or receiving treatment for inflammation Outcomes: potential effect of interaction between inflammation and CYP activity, -Study design: clinical trials and case reports/series.

### Database and Search Strategy

The literature search was performed in PubMed via MEDLINE, the database of biomedical publications, for studies and case reports/series until January 7, 2021. To expand it, we also performed a manual search of references for potentially relevant articles. The keywords used were “inflammation”, “cytochrome P450”, “cytochromes P450” and “CYP450.”

### Study Selection

We applied the eligibility criteria described below in order to filter relevant publications from the total of results provided by the literature search.

The types of studies included in our literature search were randomized controlled trials, non-randomized studies, and observational studies, including case reports and series, published as full-text articles and congress abstracts in English. The year of publication selected was from database inception until January 7, 2021. Study participants had to be older than 18 years old, including healthy subjects and patients with an inflammatory condition, caused by disease, treatment or a medical or surgical procedure. The outcomes of interest were the effect of potential inflammation (suggested or provided) on metabolic ratios (MR) of CYP isoforms, the PK/PD and the safety profile of CYP substrates.

**FIGURE 1:**
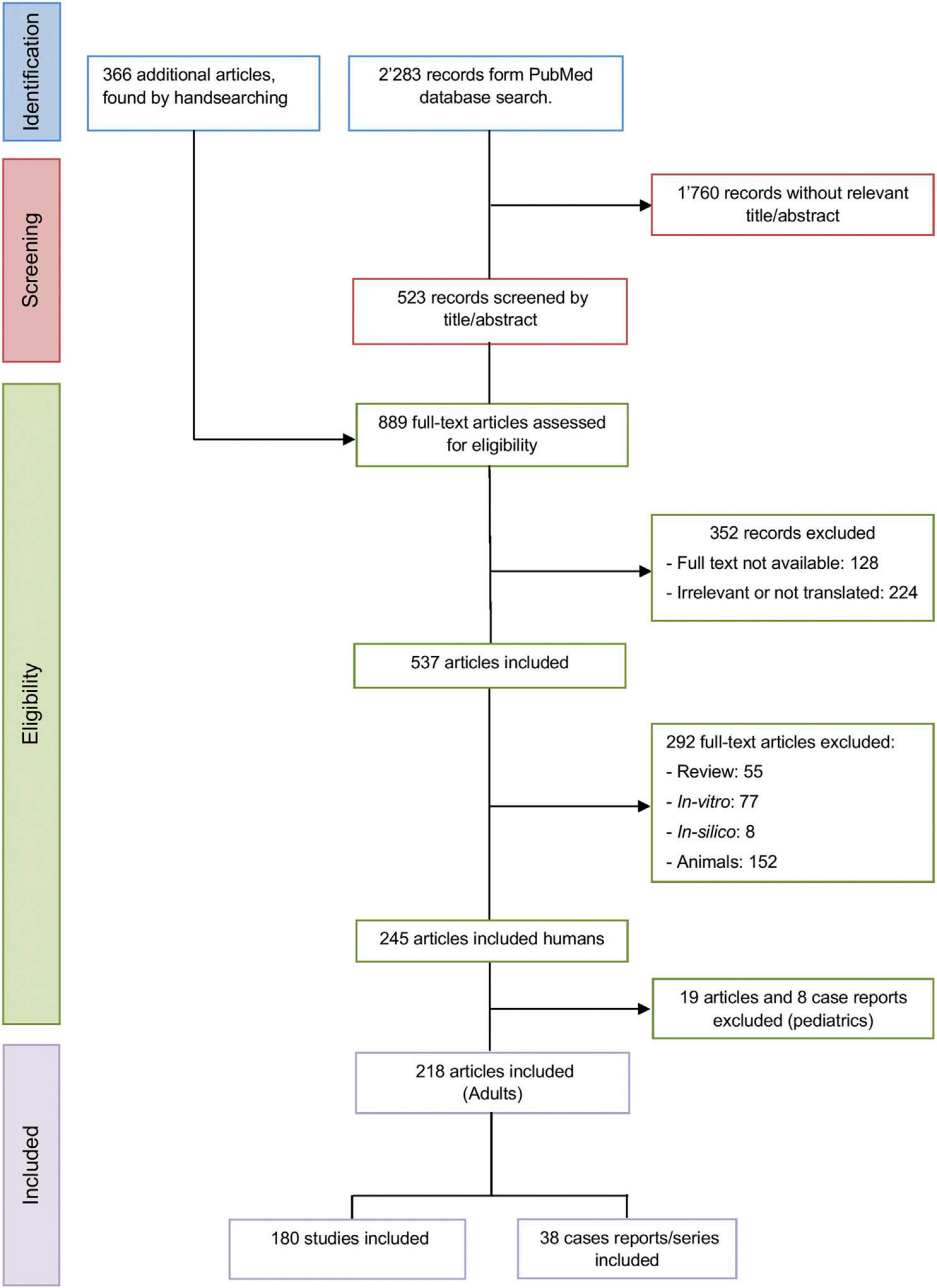
PRISMA flowchart of the studies selection process.

Successive steps in article selection included reading the title, abstract and full text according to the predefined eligibility criteria to screen for potentially relevant records. The selected articles were classified into literature reviews and *in-vitro*, animal, *in-silico* and human studies. Then, only studies involving adults (defined as over 18 years old) were kept, classified into studies or case reports/series. The same procedure was applied to assess the inclusion of additional articles identified by the manual search. The study selection process was summarized in a flowchart created according to the PRISMA statement requirements (Figure 1) (Moher et al., 2009).

### Data Extraction and Management

Articles selected from the search results were collected and exported to the reference management software Zotero (version 5.0.85, ^©^ 2006–2018 Contributors) and merged to remove duplicates. Data from the included articles were extracted and synthetized. The authors extracted the following data according to the PICOS framework discussed above. These included study design, sample size, source of inflammation and comparators, victim drugs and CYP involved, and outcomes of interests (potential effect of interaction). When a CYP substrate was used in the article to determine whether or not inflammation or concomitant drugs altered its PK/PD profile, a verification of its metabolic pathway was performed. The verification process was performed using the Summary of Product Characteristics (SmPCs), the Lexi-Interact drug interaction checker and the Geneva table of CYP substrates, inhibitors, and inducers (Uptodate,; Samer et al., 2013).

## Results

### Identification and Selection of the Studies

The primary search, performed in PubMed, yielded a total of 2′283 articles that were screened according to their title and abstract. Of the remaining 523 articles, an additional 366 articles were identified by cross-referencing and handsearching of the reference list of the relevant articles (*n* = 889). Of these, 352 records were removed because the full text was not available (*n* = 128) or because they were considered irrelevant or not translated into English (*n* = 224). The remaining 537 articles were classified into review articles (*n* = 55), *in-vitro* (*n* = 77) or *in-silico* (*n* = 8) studies, and animal (*n* = 152) or human (*n* = 245) studies. The articles and case reports concerning the pediatric population (*n* = 27) are the subject of another systematic review and were excluded from this work (Lenoir et al., 2021). Finally, 218 articles conducted in adults were included and classified into studies (*n* = 180) and case reports/series (*n* = 38) for analysis (Figure 1).

### Results of the Studies

**FIGURE 2:**
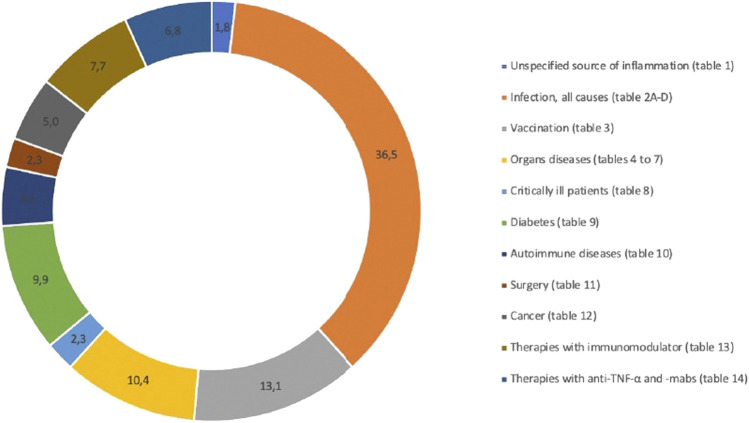
Distribution (%) of included references according to the different sources of inflammation.

The 218 eligible publications are summarized in Table 1 through 14. The drug-disease interactions found in the selected articles were divided into fourteen different sources of inflammation: unspecified source of inflammation (Table 1), infection (Table 2A), infection-example hepatitis (Table 2B), infection-example HIV (Table 3C), infection-example SARS-CoV-2 (Table 2D), vaccination (Table 3), kidney disease (Table 4), liver disease (Table 5), lung disease (Table 6), heart disease (Table 7), critically ill patients (Table 8), diabetes (Table 9), autoimmune diseases (Table 10), surgery (Table 11), cancer (Table 12), therapies with immunomodulator (Table 13) and therapies with anti-TNF-α and -mabs (Table 14). The most cited inflammation perpetrator was infection and the most studied CYP was CYP3A. CYP3A subfamilies refers to CYP3A4 and CYP3A5, because the probe drugs used to assess the activity of CYP3A4 are metabolized by these two isoenzymes and no distinction can be made between them. Distribution in percent of all the references in the different categories are illustrated in Figure 2.

**TABLE 1:** Impact of unspecified source inflammation on CYP substrates, explained totally or partially by modulation of CYP activity.

**Inflammation characterized by**	**Victim drugs (CYPs concerned)**	**Number of subjects**	**Potential effect of interaction**	**References and design**
IL-10 injection	tolbutamide (CYP2C9), caffeine (CYP1A2), dextromethorphan (CYP2D6) and midazolam (CYP3A)	12	- significantly but moderately decreased CYP3A4 activity (12 ± 17%, *p* < 0.02)	Wienkers and Heath (2005) Double-blind crossover study
			- significantly increased CYP2C9 activity (38 ± 25%, *p* < 0.005), - no significant changes in either CYP1A2 or 2D6 activity	
Elevated CRP levels (>1.5 mg/dl)	perampanel (CYP3A4)	111 = Total 23 = CRP>1.5 mg/dl 13 = enzyme-inducing AEDs 10 = no enzyme-inducing AEDs	- perampanel C/D increased by 53.5 and 100.8% respectively when CRP >1.5 mg/dl	Lynch and Price (2007) Cohort study
			- correlation between serum CRP level and C/D of perampanel (*r* = 0.44, *p* < 0.001)	
Erythrocyte sedimentation rate (ESR) > 20 mm vs. control	Oxprenolol (CYP2C9, 2D6, 3A4 and 1A2 substrate)	18	- mean oxprenolol AUC 2-fold greater in inflammation group	Zhou et al. (2017) Cohort study
CRP serum levels	tacrolimus (CYP3A4)	31-year-old man	-tacrolimus C/D increased during two inflammation episodes by 54% (cholestasis) and 141% (infection following surgery), and strongly correlated with CRP (r2 = 0.78, *p* = 0.079)	Wilkinson (2005) case report

**TABLE 2A:** Impact of infection on CYP substrates, explained totally or partially by modulation of CYP activity.

**Inflammation characterized by**	**Victim drugs (CYPs concerned)**	**Number of subjects**	**Potential effect of interaction**	**References and design**
Lipopolysaccharides (LPS)-induced inflammation	theophylline (CYP1A2), hexobarbital (CYP2C19) and antipyrine (CYP1A2, 2B6, 2C8, 2C9, 2C18 and 3A4)	12	- significant repression of CYPs activity (takes several hours to develop)	Kato (2020), Crossover study
Two injections of Gram-negative bacterial endotoxin	theophylline (CYP1A2), hexobarbital (CYP2C19) and antipyrine (CYP1A2, 2B6, 2C8, 2C9, 2C18 and 3A4)	9	- significant decrease of clearances of all probes compared with the saline control studies, - endotoxins injections associated with decreased hepatic drug metabolism, mainly CYP1A2 and 2C19	Food and Drug Administration, Cross-over clinical trial
Administration of a single oral dose of 10 mg/kg of etiocholanolone	antipyrine (CYP1A2, 2B6, 2C8, 2C9, 2C18 and 3A4)	14 = significant fever (fever index >50)	- half-life was significantly prolonged (29.3%, *p* < 0.005) in patients with significant fever	de Jong et al. (2020)
		19 = failed to develop significant fever (fever index <50)	- no significant change of half-life (*p* > 0.8) in patients without significant fever	Cross-over clinical trial
			- no correlation between the magnitude of fever and the extent to which half-life was prolonged	
Acute pneumonia	antipyrine (CYP1A2, 2B6, 2C8, 2C9, 2C18 and 3A4)	14	- 1.5 fold increased clearance 14 and 28 days after the acute illness	Stanke-Labesque et al. (2020)
			- enhancement of clearance in 28 days represented a 36% improvement	Cohort study
Liver fluke infection (uninfected, infected only and infected with fibrosis)	coumarine (CYP2A6)	- Total = 91	- 26% lower urine levels of 7-hydroxycoumarine (7-HC) after praziquantel (*p* < 0.001) compared to initial assessment	Stavropoulou et al. (2018)
		- 73 completed the two assessments	- infected individuals excreted slightly higher levels of 7-HC in the 0–2 h period	Cohort study
Herpes zoster	warfarin (CYP2C9)	66-year-old woman	- acute spinal subdural hematoma and subarachnoid haemorrhage during the course of a thoracic level infection	Germolec et al. (2018)
			- 3-fold increased PT times requiring vitamin K administration	Case report
Visceral leishmaniasis	midazolam (CYP3A), omeprazole (CYP2C19), losartan (CYP2C9)	24	- significantly increased midazolam CL/F (*p* = 0.018) 2–3 days and 3–6 months after curative chemotherapy	Gabay and Kushner (1999)
			- significantly increased omeprazole CL/F (*p* = 0.008) 2–3 days and 3–6 months after curative chemotherapy	Cohort study
			- CYP2C9 activity not significantly different between	
Influenza A	theophylline (CYP1A2)	50-year-old woman	- toxicity symptoms after infection	Morgan (2001)
			- increased theophylline levels (1.5x above normal values)	Case report
Acute illness	theophylline (CYP1A2)	3	- 2-fold or 3-fold variation in clearance during acute illness	Morgan (1997)
			- clearance decreased during worsening of airway obstruction in one patient	Case series
			- 2 patients had increased clearance during the improvement of their condition (pneumonia and congestive heart failure)	
Elevated CRP levels (>5 mg/L) vs control	citalopram (major CYP2C19, minor CYP3A4) and venlafaxine (major CYP2D6, minor CYP3A4 and 2C19)	15 citalopram	- no statistical differences in citalopram and venlafaxine concentrations or in MR of both drugs in samples with elevated CRP levels	Liptrott and Owen (2011)
		39 venlafaxine		Cohort study
Elevated serum levels of CRP	risperidone (bioactivated by CYP3A4 and CYP2D6)	2 females (56 and 38 years old)	- close temporal association between serum levels of risperidone active moiety (risperidone + 9-hydroxyrisperidone) and CRP	Renton (2005)
			- > 3x increase of C/D during elevated CRP serum concentration	Case report
			- parallel fluctuation of drug levels and CRP which necessitated dose adjustments, but the MR was unchanged, suggesting that the CYP2D6-catalyzed formation of 9-hydroxyrisperidone was not affected	
Pneumonia	risperidone (bioactivated by CYP3A4 and CYP2D6)	56-year-old man	5-fold higher risperidone dose requirement during pneumonia	Ruminy et al. (2001)
				Case report
Elevated serum levels of CRP (>5 mg/L)	clozapine (CYP1A2), quetiapine (CYP3A4 and CYP2D6) and risperidone (CYP3A4 and CYP2D6)	33 clozapine, 32 quetiapine 40 risperidone	- C/D of clozapine was significantly higher (*p* < 0.01) and CYP1A2 MR (NCLZ/CLZ) significantly lower (*p* < 0.05)	Moher et al. (2009)
			- positive and significant correlation between clozapine and CRP levels (*r* = 0.313, *p* < 0.01)	Cohort study
			- no difference in C/D or in MR of quetiapine	
			- C/D of risperidone was significantly higher (*p* < 0.01) and MR decreased (NS)	
Elevated serum levels of CRP	clozapine (CYP1A2)	27 high drug level	mean CRP value significantly higher (*p* = 0.005) in patients with elevated clozapine level	Uptodate
		36 normal drug level		Case-control study
Elevated serum level of CRP of 130 mg/L	clozapine (CYP1A2)	44-year-old man	- admission to hospital because of symptoms of clozapine toxicity	Samer et al. (2013)
			- elevated clozapine levels	Case report
			- condition improved when treatment was discharged	
Elevated serum level of CRP of 256 mg/L	clozapine (CYP1A2)	50-year-old man	- 5-fold increased plasma levels 4 days after admission	Lenoir et al. (2021)
				Case report
Sepsis	clozapine (CYP1A2)	61-year-old woman	- clozapine toxicity symptoms	Luong et al. (2016)
			- increased clozapine serum levels = 4318 ng/ml (References = 350–700 ng/ml)–All patients improved after dose reductions	Case reports
Suspected infections	clozapine (CYP1A2)	4	- clozapine toxicity symptoms in usually stable patients	Dote et al. (2016)
			- patients improved after dose reduction or therapy discontinuation	Case series
Suspected infections	clozapine (CYP1A2)	62-year-old man	- clozapine levels increased during infection (from 377 ng/ml to 1′628 ng/ml)	Encalada Ventura et al. (2015)
				Case report
Respiratory infection	clozapine (CYP1A2)	34-year-old man	- increased clozapine levels to 1245 ng/ml during infection	Niioka et al. (2017)
				Case report
Lung abscess	clozapine (CYP1A2)	29-year-old man	- increased clozapine levels during infection (from 681 ng/ml to 1′467 ng/ml)	Encalada Ventura et al. (2015)
			- No signs of clozapine toxicity	Case report
Influenza A	clozapine (CYP1A2)	33-year-old woman	- increased clozapine levels during infection (from 661 ng/ml to 1′300 ng/ml)	Encalada Ventura et al. (2015)
			- symptoms of clozapine toxicity	Case report
Pneumonia	clozapine (CYP1A2)	42-year-old man	- increased clozapine levels during infection (from 1′024 ng/ml to 2′494 ng/ml)	Encalada Ventura et al. (2015)
			- symptoms of clozapine toxicity	Case report
Pneumonia	clozapine (CYP1A2)	35-year-old man	- increased median clozapine C/D ratios at the peak of infection	Vreugdenhil et al. (2018)
				Case report
Upper respiratory tract infection	clozapine (CYP1A2)	68-year-old woman	- increased clozapine levels during infection (peaked at 1′096 ng/ml)	van Wanrooy et al. (2014)
			- toxicity symptoms	Case report
Upper respiratory tract infection	clozapine (CYP1A2)	47-year-old man	- On day 24 and 25 (highest level of infection severity), serum concentration levels increased to 881.2 and 663.5 ng/ml, respectively	Schulz et al. (2019)
				Case report
Urinary tract infection	clozapine (CYP1A2)	51-year-old woman	- increased clozapine levels during infection (peak at 1′066 ng/ml)	Veringa et al. (2017)
			- patients improved after dose reduction and recovery	Case report
Urinary tract infection	clozapine (CYP1A2)	45-year-old woman	- increased clozapine levels during infection (from 705 ng/ml to 2′410 ng/ml)	Encalada Ventura et al. (2015)
			- toxicity symptoms	Case report
Urinary tract infection	clozapine (CYP1A2)	62-year-old man	- increased clozapine levels during infection (from 432 ng/ml to 1′192 ng/ml)	Encalada Ventura et al. (2015)
			- no toxicity symptoms	Case report
Urinary tract infection	clozapine (CYP1A2)	64-year-old woman	- decreased clozapine levels after infection recovery (from 749.4 to 260.0 ng/ml)	Gautier-Veyret et al. (2019)
			- toxicity symptoms	Case report
Infections	clozapine (CYP1A2)	16 patients with 18 episodes	- only 2 episodes did not require any relevant changes of dosage	Bolcato et al. (2021)
				Case series
Infections	clozapine (CYP1A2)	3	- clozapine toxicity symptoms	Elin et al. (1975)
			- 2.5-7-fold increased clozapine serum concentration during infections	Case series
Diarrheic stools and gastrointestinal bacterial infection	clozapine (CYP1A2)	23 years old man	- at admission, CRP serum concentration = 130 mg/ml and clozapine serum concentration = 9074 nmol/L (References interval 200–2500 nmol/L)	Blumenkopf and Lockhart (1983)
			- 1 month before, serum concentration = 1919 nmol/L 1 month before admission and fairly constant during the last years	Case report
Bacterial pneumonia	clozapine (CYP1A2)	53-year-old woman	- trough concentration = 2074 μg/L at day 0 (before any antibiotics treatments)	Khan and Khan (2019)
			- previous trough concentrations were three times lower	Case report
			- during the infection, CRP = 152 mg/L and α1-glycoprotein = 2398 mg/L	
			- concentration decreased nearly to the previous levels after 2 weeks (624 ± 214 mg/L)	
Increased CRP level	voriconazole (CYP3A4 and CYP2C19)	63	- increased CRP levels associated with significantly increased voriconazole C/D (*p* < 0.05)	Vozeh et al. (1978)
			- CYP3A4 and CYP2C19 downregulated by inflammation	Retrospective study
				Cohort study
Increased CRP level	voriconazole (CYP3A4 and CYP2C19)	19	- inflammatory response positively associated with voriconazole concentration (*r* = 0.62, *p* < 0.001)	Leung et al. (2014)
			- inflammatory response negatively associated with voriconazole MR (rho = -0.64, *p* < 0.001)	Cohort study
Elevated CRP level	voriconazole (CYP3A4 and CYP2C19)	54	- voriconazole/N-oxide ratio could be predicted by the CRP concentration with a standardized regression coefficient of 0.380 (*p* = 0.001)	Haack et al. (2003)
				Cohort study
Elevated IL-6, IL-8 and CRP levels	voriconazole (CYP3A4 and CYP2C19)	22	- correlation between IL-6 (*r* = 0.46, *p* < 0.0001), IL-8 (*r* = 0.42, *p* < 0.0001) and CRP (*r* = 0.53, *p* < 0.0001) and trough concentration	de Leon and Diaz (2003)
				Cohort study
CRP serum level	voriconazole (CYP3A4 and CYP2C19)	Total = 128	- trough concentration increased by 0.015 mg/L every 1 mg/L increase in CRP	Jecel et al. (2005)
- Elevated (>200 mg/L)			- correlation between trough concentration and CRP levels (*p* < 0.001), and with severity of inflammation	Retrospective study
- Moderate (>41 mg/L, <200 mg/L)				Cohort study
- Control (<40 mg/L)				
Multiple infections along his 5 months hospital stay	voriconazole (CYP2C19 and 3A4), meropenem and their combinations	78-year-old man	- decreased voriconazole dose requirements	Darling and Huthwaite (2011)
				Case report
CRP serum level	voriconazole (CYP3A4 and CYP2C19)	34	- MR significantly decreased with higher CRP concentration after adjustment (*p* < 0.001)	Espnes et al. (2012)
		20 = patients with CYP2C19 genotype performed	- extent of decrease of MR and increase of trough concentration varied between the different genotypes (*p* < 0.001 and *p* = 0.04, respectively)	Prospective study
CYP2C19 genotype				Cohort study
CRP serum levels	voriconazole (CYP3A4 and CYP2C19) and itraconazole (CYP3A4)	41 voriconazole	- C/D of voriconazole and of voriconazole N-oxide positively (r = 0.61, *p* < 0.01) and negatively (r = -0.52, *p* < 0.01) correlated with CRP levels, respectively	Raaska et al. (2002)
		42 itraconazole	- C/D of itraconazole (*p* = 0.33) and its hydroxide (*p* = 0.52) were not correlated with CRP	Cohort study
CRP serum levels	voriconazole (CYP3A4 and CYP2C19)	31 = with overdose	- mean CRP level significantly higher (*p* < 0.0001) in patients who experienced an overdose (188 mg/L) compared to those who did not (37 mg/L)	Levine and Jones (1983 1)
		31 = without overdose	- patients with CRP levels >96 mg/L (median level) had a 27-fold higher risk of overdose than patients with CRP levels <96 mg/L	Case-control study
Inflammation level	voriconazole CYP2C19 and 3A4)	64-year-old man	- voriconazole C/D associated with inflammation level	Clark et al. (2018)
				Case report
Influenza-like illness	phenytoin (CYP2C9 and CYP2C19 substrates and induces CYP2C9, 2C19 and 3 A)	52-years-old woman	- became increasingly drowsy, moody, complaining of staggering, difficulty to talking and visual disturbance with toxic phenytoin levels (51 μg/ml)	Kwak et al. (2014)
				Case report
Pneumonia	perampanel (CYP3A4)		- 3.5-fold increase perampanel concentrations, - reversible within 7 days after CRP normalization	Lynch and Price (2007))
				Case report
Inoculation of Malaria	quinine (CYP3A4)	5	- increase quinine MR during infection (*p* < 0.01)	Takahashi et al. (2015)
				Cross-over study
Infection disease state (pneumonia, endocarditis, wound infection or gastroenteritis) vs healthy state	bisoprolol (CYP2D6 and 3A4) and nitrendipine (CYP3A4)	20	- PK parameters of bisoprolol unchanged (*p* > 0.05)	Hefner et al. (2016)
			- bioavailability of S-enantiomer twice that of R-nitrendipine in infection (*p* < 0.01)	Cohort study
			- 2-fold increased AUC and Cmax of S-nitrendipine (*p* = 0.010 and *p* = 0.012 respectively) and R-nitrendipine (*p* = 0.005 and *p* = 0.029)	
Enteritis with diarrhoea	tacrolimus (CYP3A)	52	- mean tacrolimus trough level 2.3 times higher during enteritis (*p* = 0.0175)	Pfuhlmann et al. (2009)
			- mean trough level returned to their baseline levels 2 weeks after onset	Cohort study
Helicobacter pylori infection in cirrhotic patients	/	21 tested positive and 11 not	Hp-infected cirrhotic patients had a significant lower mean of the monoethylglycinexylide (MEGX) test compared to non-infected patients (*p* = 0.006), while 13C-galactose breath test (GBT) was not	Abou Farha et al. (2012)
				Case-control study
Sepsis	tacrolimus (CYP3)	41-year-old man	151% increased tacrolimus C/D during sepsis	Wilkinson (2005)
				Case report
Dermatitis	clozapine (CYP1A2)	57-year-old woman	- On days 36 and 43 (highest level of dermatitis severity), clozapine serum concentration increased to 889.2 and 1′012 ng/ml, respectively	Schulz et al. (2019) Case report

**TABLE 2B:** Impact of hepatitis on CYP substrates, explained totally or partially by modulation of CYP activity.

**Inflammation characterized by**	**Victim drugs (CYP concerned)**	**Number of subjects**	**Potential effect of interaction**	**References and design**
Chronic hepatitis C	antipyrine (CYP1A2, 2B6, 2C8, 2C9, 2C18 and 3A4)	12 = chronic hepatitis C	- decreased clearance and greater excretion in urine (about 50%, *p* < 0.01)	ten Bokum et al. (2015)
		18 = controls	- no difference in hepatic enzymes levels but Child Pugh Score correlated with clearance (*r* = −0.73, *p* = 0.007)	Case-control study
Chronic hepatitis C	antipyrine (CYP1A2, 2B6, 2C8, 2C9, 2C18 and 3A4)	85	- no difference in clearance before and after 6 weeks of interferon treatment	Ruan et al. (2017)
			- 14% clearance increased (*p* < 0.05) 6 months later among responders but not in those who had failed to respond to interferon	Cohort study
Acute viral hepatitis	antipyrine (CYP1A2, 2B6, 2C8, 2C9, 2C18 and 3A4)	6	- decreased plasma half-life and plasma clearance during the acute phase of hepatitis compared to recovery period (*p* < 0.02)	Ruan et al. (2018)
				Cohort study
Acute hepatitis	hexobarbital (CYP2C19)	13 = hepatitis	- decreased elimination half-life in patients with hepatitis compared to controls (490 ± 186 min vs. 261 ± 69 min, *p* < 0.001)	Ruan et al. (2020)
		14 = controls		Case-control study
Hepatitis C infection (IFN)	Cyclosporin A (CyA) and tacrolimus (CYP3A4)	26 = hepatitis C infection	- Lower doses (*p* < 0.05) in hepatitis C as compared to controls, while levels were comparable	Sonne et al. (1985)
		78 = controls		Case-control study
Acute viral hepatitis C	CyA (CYP3A4)	18 = HCV Ab +	- CyA levels significantly higher in HCV Ab + (*p* = 0.0001)	Satarug et al. (1996)
		18 = HCV Ab -		Case-control study
Acute viral hepatitis C	CyA (CYP3A4)	11 = anti-HCV +	- altered CyA PK (higher peak levels and drug exposure) in HCV+, especially those with viremia	Hanada et al. (2012)
		11 = controls		Case-control study
Acute viral hepatitis C	CyA (CYP3A4)	10 = anti-HCV +	- CyA AUC 69% (*p* < 0.01) and 32% (*p* < 0.01) higher in pre- et post-transplant studies in HCV + patients	Hanada et al. (2012)
		14 = controls		Case-control study
Acute viral hepatitis	meperidine (CYP2B6, 2C19 and 3A4)	14 = acute viral hepatitis	- terminal plasma half-life significantly prolonged in acute viral hepatitis compared to controls (*p* < 0.001) and 2-fold change in total plasma clearance observed (*p* < 0.002)	Latorre et al. (2002)
		15 = controls		Case-control study
Acute viral hepatitis	meperidine (CYP2B6, 2C19 and 3A4)	5	- total plasma clearance increased from 488 ± 132 ml/min to 1200 ± 555 ml/min and the terminal half-life decreased from 8.24 ± 3.71 to 3.25 ± 0.80 h respectively (*p* < 0.005)	Latorre et al. (2002)
			- values after recovery were not significantly different from those of the control group	RCT
Chronic hepatitis C (CHC)	midazolam (CYP3A4)	107 = controls	- MR decreased by 37 and 54% (*p* < 0.05) in patients with hepatitis C treatment-naive and interferon null-responders respectively, compared to controls	Tuncer et al. (2000)
		35 = CHC naïve to treatment	- consistent reductions in CYP3A4 activity between healthy volunteers and patients infected, most substantial difference with interferon null-responders	Case-control study
		24 = CHC null responders to IFN		
liver kidney microsome type 1 (LKM-1) antibodies	dextromethrophan (CYP2D6)	10 negative and 10 positive patients for LKM-1	- dextromethorphan-to-dextrorphan (DEM/DOR) ratio was significantly higher in liver kidney microsome type (LKM-1) positive patients (*p* = 0.004), showing that CYP2D6 activity had decrease (antibodies are targeted against CYP2D6)	Wolffenbüttel et al. (2004)
				Case-control study
Hepatitis A	coumarine (CYP2A6)	9 = hepatitis A	- mean reduction of 37% (*p* < 0.05) of the total urine excretion	McHorse et al. (1975)
		20 = controls	- CYP2A6 lower metabolic activity in hepatitis patients	Case-control study
Hepatitis C virus (HCV) vs control	omeprazole (CYP2C19) and cortisol (CYP3A)	31 = HCV (9 with chronic hepatitis and	- mean omeprazole hydroxylation index in HCV patients were significantly higher compared with healthy subjects, with lower CYP2C19 activity	Smolders et al. (2017)
		22 with cirrhosis)	- mean clearance of cortisol decreased significantly (*p* < 0.001) in CLD patients	Case-control study
		30 = controls		
Chronic HCV treated with sofosbuvir	tacrolimus (CYP3A)	56-year-old male	- through concentration decreased after initiation of HCV treatment that required an increase of dosage	Kawaoka et al. (2016)
		74-year-old male		Case report
HCV treated with daclatasvir/asunaprevir	tacrolimus (CYP3A)	57-year-old man	- case 1: slight increase in trough blood concentration after the start of the combination therapy but no dose adjustment	Saab et al. (2016)
		63-year-old man	- case 2: through blood concentration decreased after the start of the combination therapy and dosage was increased	Case report
HCV before and after treatment	tacrolimus (CYP3A) and cyclosporine (CYP3A)	52	- statistically significant difference in daily dose adjusted per weight or serum levels of tacrolimus after achieving a sustained viral response	Raschzok et al. (2016)
			- no statistically significant difference in daily dose adjusted per weight or serum levels of cyclosporine after achieving a sustained viral response	Cohort study
HCV treated with directly acting antivirals	tacrolimus (CYP3A) and^13^C-methacetin (LiMAx test, CYP1A2)	21	- mean LiMAx increased from 344 ± 142 to 458 ± 170 μg/kg/h between the start of treatment and week 12 (*p* < 0.001) (value in healthy volunteers = 430 ± 86 μg/kg/h)	Ueda and Uemoto (2016)
			- tacrolimus C/D decreased over the same period (*p* = 0.0017)	Cohort study
HCV treated with daclatasvir/asunaprevir	tacrolimus (CYP3A)	10	- C/D ratio decreased from 3.95 ng/ml per mg to 2,975 ng/ml per mg after 2 weeks of administration	van den Berg et al. (2001)
				Cohort study
HCV	tacrolimus (CYP3A)	7 = HCV	- dose required to obtain therapeutic levels was comparable in the 2 groups during the first 3 weeks	Kugelmas et al. (2003)
		13 = transplanted for other indications	- dose requirement decreased sharply in HCV patients (20% of the value in controls)	Cohort study
			- dose requirement increased by more than 50% in 2 patients treated with IFN-α/ribavirin	
HCV treated with anti-HCV therapy	tacrolimus (CYP3A) and cyclosporine (CYP3A)	12 (7 cyclosporine and 5 tacrolimus) = responders	- cyclosporine and tacrolimus levels at baseline vs after HCV RNA negativation decreased significantly (*p* = 0.018 for cyclosporine and *p* = 0.044 for tacrolimus)	Ueda et al. (2015)
		18 (7 cyclosporine and 11 tacrolimus) = non-responders	- cyclosporine and tacrolimus levels in non-responders did not change between baseline and the end of anti-HCV therapy (*p* = 0.24 for cyclosporine and *p* = 0.32 for tacrolimus)	Cohort study
HCV treated with simeprevir	tacrolimus (CYP3A) and cyclosporine	2	- C/D ratio of calcineurin inhibitors were elevated in the first 2 weeks in both cases, but decreased thereafter, necessitating an increase in the dose	Morcos et al. (2013)
				Case report

**TABLE 2D:** Impact of SARS-CoV-2 on CYP substrates, explained totally or partially by modulation of CYP activity.

**Inflammation characterized by**	**Victim drugs (CYP concerned)**	**Number of subjects**	**Potential effect of interaction**	**References and design**
SARS-CoV-2 and treatment with tocilizumab	lopinavir/ritonavir (CYP3A) and hydroxychloroquine (CYP2D6)	41 = without tocilizumab, 51 = tocilizumab (35 before and 16 after)	- lopinavir concentrations positively correlated with CRP (*r* = 0.37, *p* < 0.001) and significantly lower after tocilizumab, - no correlation between CRP and hydroxychloroquine plasma concentration	Marzolini et al. (2020), Cohort study
SARS-CoV-2 vs. HIV-patients	lopinavir/ritonavir (CYP3A)	12	- lopinavir trough concentration in patients with SARS-CoV-2 infection were significantly higher than those usually observe in HIV-infected patients (18′000 vs. 5365 ng/ml)	Gregoire et al. (2020), Cohort study
SARS-CoV-2	clozapine (CYP1A2)	38-year-old-man	- symptoms of clozapine toxicity, - clozapine level increased by 0.57–0.73 mg/L and norclozapine increased by 0.22 mg/L to 0.31 mg/L after SARS-CoV-2 infection	Cranshaw and Harikumar (2020), Case report
SARS-CoV-2	lopinavir/ritonavir (CYP3A)	8	- through concentration associated with CRP level (*r* = 0.81, p = unknown), - through levels were 2-fold higher in patients with SARS-CoV-2 infection than HIV patients	Schoergenhofer et al. (2020), Cohort study
SARS-CoV-2	apixaban (CYP3A), rivaroxaban (CYP3A), edoxaban (CYP3A)	5 = apixaban, 3 = rivaroxaban, 3 = edoxaban	- alarming increase in DOAC plasma levels compared to pre-hospitalization levels, - possible role of concomitant drugs (CYP3A inhibitors) or disease-related organ dysfunctions	Testa et al. (2020), Cohort study
SARS-CoV-2 vs HIV-patients	darunavir (CYP3A)	30 = SARS-CoV-2 25 = HIV	- median CL/F was significantly lower in SARS-CoV-2 patients with IL-6 levels >18 pg/ml than <18 pg/ml or HIV patients (*p* < 0.0001), - increasing level of IL-6 affected concentration vs time simulated profile	Cojutti et al. (2020), Case-control study

**TABLE 3C:** Impact of HIV on CYP substrates, explained totally or partially by modulation of CYP activity.

**Inflammation characterized by**	**Victim drugs (CYP concerned)**	**Number of subjects**	**Potential effect of interaction**	**References and design**
AIDS patients vs control	clindamycin (CYP3A)	16 = AIDS	- clearance values normalized to subject body weight were 0.27 ± 0.06 L/h/kg for the healthy volunteers and 0.21 ± 0.06 L/h/kg for the AIDS patients (*p* = 0.014)	Breimer et al. (1975)
		16 = healthy volunteers	- ADR following administrations (same dose) were observed in eight patients with AIDS	Case-control study
HIV-infected patients vs control	midazolam (CYP3A), dextromethorphan (CYP2D6) and caffeine (CYP1A2)	17 = HIV-infected	- midazolam clearance was significantly lower in HIV-infected patient compared with healthy volunteers (CI95% = 0.68–0.92) and a significant relationship was found with TNF-α (*r* = −0.66, *p* = 0.008)	Imai et al. (2011)
		17 =	- urinary dextrometorphan MR was significantly higher in HIV-infected patients than in healthy volunteers (CI95% = 2.36–42.48) and a trend was observed for an association with the increase in TNF-α concentration (*r* = 0.49, *p* = 0.06)	Case-control study
		uninfected	- caffeine metabolism was no significantly different in HIV-infected subjects compared to non-smokers healthy volunteers (controlled for smoking status) (CI95% = 0.83–3.11)	
HIV-infected patients vs control	midazolam (CYP3A) and	30 = HIV-infected	- CYP3A4 activity in HIV infected patients was approximately 50% of the activity in healthy volunteers but it was mainly attributable to a lower intestinal CYP3A4 activity, while hepatic CYP3A was not different	Gatti et al. (1993)
	dextromethorphan (CYP2D6)	12 = healthy volunteers	- CYP2D6 activity was essentially comparable	Case-control study
HIV-positive patients	dextromethorphan (CYP2D6)	61	- 2 of the 59 patients with an NM genotype expressed a PM phenotype and 4 NM genotype patients were less extensive dextrometorphan metabolizers than any of the patients receiving medication known to inhibit CYP2D6	Jones et al. (2010)
				Cohort study
HIV-1 infected patients vs control	darunavir (CYP3A)	Unknown, information obtained from Summary of Product Characteristics (SmPC)	- exposure to darunavir was higher in HIV-1 infected patients	Jetter et al. (2010)
			- explained by the higher concentrations of α1-glycoprotrein in HIV-1 infected patients, resulting in higher darunavir binding to plasma AAG and, therefore, higher plasma concentrations	Case-control study
HIV-infected patients vs healthy volunteers	saquinavir (CYP3A)	33 = HIV-infected	- co-administration of ketoconazole increased saquinavir AUC by 190 and 69% in healthy volunteers and HIV-infected patients, respectively while co-administration of rifampicin decreased saquinavir area under the curve by 70 and 46%	European medicines agency
				Case-control study
		12 and 14 = control		
HIV-infected patients vs healthy controls	atazanavir and atazanavir with ritonavir (CYP3A)	Unknown, information obtained from SmPC	- mean AUC of atazanavir and atazanavir with ritonavir were 29′303 and 61′435 ng*h/mL respectively in healthy volunteers, vs. 22′262 and 53′761 ng*h/ml, respectively in HIV-infected patients	Grub et al. (2001)
				Case-control study
HIV-infected patients vs healthy controls	lopinavir with ritonavir (CYP3A)	Unknown, information obtained from SmPC	- no substantial differences observed between the two groups	Packageinserts
				Case-control study
HIV-infected patients vs healthy controls	atazanavir (CYP3A)	10 = HIV-infected	- mean atazanavir AUC in HIV-infected patients was 14′187 ng*h/ml compared with 33′097 ng*h/ml in healthy volunteers	Le Tiec et al. (2005)
		36 = healthy volunteers	- after 14 and 20 days of atazanavir in HIV patients and healthy volunteers, respectively, AUC were 46′073 and 57′039 ng*h/ml	Case-control study
Patients with different stage of HIV infection vs control	caffeine (CYP1A2)	29 = AIDS	- metabolic status was not change in HIV asymptomatic patients but changed in AIDS patients (with acute illnesses or stable)	Venuto et al. (2018)
		29 = AIDS-stable		Case-control study
		18 = HIV-infected		
		29 = control		
HIV infected patients	atazanavir (CYP3A)	107 = HIV-1 infected	- apparent oral clearance was not significantly correlated with inflammatory biomarkers	Lee et al. (1993)
				Cohort study

**TABLE 3:** Impact of vaccination on CYP substrates, explained totally or partially by modulation of CYP activity.

**Inflammation characterized by**	**Victim drugs (CYP concerned)**	**Number of subjects**	**Potential effect of interaction**	**References and design**
Influenza vaccination	Erythromycin breath-tests (ERMBT) (CYP3A)	24 = healthy volunteers	- no significant difference between CYP3A4 activity before and 7 days after vaccination but the influenza antigen-specific production of IFN-γ by lymphocytes was highly correlated with the change in ERMBT (*r* = -0.614, *p* = 0.020) thus, IFN-γ downregulates the expression/activity of CYP3A4	Boffito et al. (2021)
				Non-random
Influenza vaccination	ERMBT (CYP3A)	15 = healthy volunteers	- significant inverse correlation between age and change in ERMBT (*r* = −0.624, *p* < 0.015) after vaccination	Stanke-Labesque et al. (2021)
				Non-random
Influenza vaccination	simvastatine (CYP3A)	68-year-old man	- hospitalized because of complaining of extreme weakness and diffuse muscle pain 5 days after influenza vaccine	Hayney and Muller (2003)
			- 24 h after the vaccination, he began to complain of diffuse myalgia and symptoms worsened	Case report
			- serum CPK value at admission was of 93′000 U/L (70 U/L 2 weeks prior to admission)	
Influenza vaccination	chloroxazone (CYP2E1)	10 = healthy volunteers	- no significant difference in the PK parameters before immunization and 7 and 21 days after vaccination	Stults and Hashisaki (1983)
				Non-random
Influenza vaccination vs controls	^13^C-aminopyrine breath test (CYP2C19, 1A2 and 3A4)	12 = vaccinated	- significant reduction (22–74%, *p* < 0.001) in aminopyrine breath test 7 days after vaccination compared to controls	Fischer et al. (1982)
		10 = controls	- metabolic activity depression was not significant 2 days after vaccination but there was still a significant reduction 21 days after vaccination	Non-random
BCG vaccination (*tuberculosis*)	theophylline (CYP1A2)	9 = patients converted to positive Mantoux skin test	- the clearance and half-life were significantly decreased and increased, respectively (*p* < 0.02), in patients with positive Mantoux skin test, as compared to controls	Stults and Hashisaki (1983)
		3 = controls		Random
Influenza vaccination	theophylline (CYP1A2)	7=3 recovering from an acute exacerbation of COPD and 4 healthy volunteers	- plasmatic concentration before and after influenza vaccination significantly increased	Goldstein et al. (1982)
				Non-random
Influenza vaccination	theophylline (CYP1A2)	13	- no difference in the mean serum theophylline levels before influenza vaccination and 24h, 72h, 1 week and 2 weeks after vaccination	Britton and Ruben (1982)
				Non-random
Influenza vaccination	theophylline (CYP1A2)	7 (chronic bronchitis and chronic airflow obstruction thus and 5 men were smokers (CYP1A2 inductor))	- no difference between the clearance rate before and 24 h after vaccination (*p* = 0.778)	Patriarca et al. (1983)
			- clearance 4–48 h after influenza vaccination was not significantly different (*p* = 0.789)	Non-random
			- serum interferon was not detected in any of the seven subjects before or 8, 16, 24, 46 h and 7–10 days following vaccination	
Influenza vaccination	theophylline (CYP1A2)	16 (COPD)	- no difference in plasma concentration 24 h before or after vaccine injection	Jackson et al. (2007)
				Non-random
Influenza vaccination	theophylline (CYP1A2)	5	- no significant variations in the serum levels before and 24 h after vaccination	Farrow and Nicholson (1984)
				Non-random
Influenza vaccination	theophylline (CYP1A2) and chlordiazepoxide (CYP3A)	8 = theophylline	- an effect of vaccination has been shown on theophylline clearance at day 1 after vaccination (*p* = 0.016) but not at day 7	MacCallum et al. (2007)
		5 = chlordiazepoxide	- no effect on chlordiazepoxide metabolism	Non-random
			- the effect seems to be greater when initial clearance is higher	
Influenza vaccination vs controls	theophylline (CYP1A2) and warfarin (CYP2C9)	152 = influenza vaccinated	- no ADR occurred in patients on theophylline in both groups and only one reaction in each group of patients who were taking warfarin	Raj et al. (1995)
		51 = unvaccinated		Case-control study
Influenza, pneumococcal, tetanus and hepatitis A vaccinations	warfarin (CYP2C9)	5′167	- not associated with INR value change	Gomolin (1986)
				Cohort study
Influenza and pneumococcal vaccination vs. controls	warfarin (CYP2C9)	25 = placebo	- no statistically significant increments in mean British Corrected Ratios for prothrombin time 2, 7- or 21-days post injections	Iorio et al. (2006)
		25 = influenza		Random
		19 = pneumococcal		
Influenza vaccination	warfarin (CYP2C9)	78	- no significant effect on anticoagulant control during the 10 days post-vaccination in the vast majority of individuals	Poli et al. (2002)
				Cohort study
Influenza vaccination	warfarin (CYP2C9)	41	- no significant difference in the mean PT 3, 7 and 14 days after vaccination for the entire group and no patient developed any major or minor bleeding episodes	Paliani et al. (2003)
				Cohort study
Influenza vaccination vs controls	warfarin (CYP2C9)	7	- no difference in the mean PT one, three and 6 weeks after vaccination	Casajuana et al. (2008)
				Cohort study
Influenza vaccination	warfarin (CYP2C9)	104	- no difference in the mean PT-INR values and mean weekly dosage between group 1 (active vaccine at day 0 and placebo at day 42) and group 2 (placebo at day 0 and active vaccine at day 42)	Kramer et al. (1984), Cross-over study
Influenza vaccination	warfarin (CYP2C9)	71 = vaccinated, 72 = controls	- no differences in the anticoagulation levels 3 months before and 3 months after the vaccination, - in the 34 vaccinated patients older than 70 years, a reduction of anticoagulation intensity was achieved in the 3 months after the vaccination and it was not the case in control group	Carroll and Carroll 2009), Case-control study
Influenza vaccination	warfarin (CYP2C9)	49 = patients, 45 = controls	- no difference in INR between patients and control groups before vaccination while 7–10 days after injection, INR significantly increased (*p* < 0.00005), - in patient group, INR increased significantly after vaccination (*p* < 0.00001)	Weibert et al. (1986), Case-control study
Influenza vaccination	225 acenocoumarol 4 warfarin (CYP2C9)	100 = intramuscular, 129 = subcutaneous	- INR decreased 24 h after intramuscular vaccination and increased in the subcutaneous group but the difference did not reach statistical significance	Plotkin et al. (2000), RCT
Influenza vaccination	warfarin (CYP2C9)	8	40% prolongation of PT (statistically significance unknown)	Pellegrino et al. (2013), Non-random
Influenza vaccination	warfarin (CYP2C9)	12 (healthy volunteers)	- no significant effect on warfarin metabolism was observed between influenza vaccination or saline injection	Pellegrino et al. (2013), Cross-over study
Influenza vaccination	warfarin (CYP2C9)	81-years-old man	- admitted with hematemesis and a 3-days history of melena and further investigations confirmed a bleeding gastric mucosa but no evidence of oesophagitis, gastritis, duodenitis or ulcer, - monthly PT had been stable and in the therapeutic ranges but the day of admission, PT was 36 s, - 10 days before admission, he received influenza vaccination. Warfarin was withheld and recovered uneventful	Pellegrino et al. (2013), Case report
Influenza vaccination	warfarin (CYP2C9)	64-years-old patient	- death from intracranial haemorrhage (INR = 15 at admission), - INR = 2 4.5 weeks before and all values over the previous 6 months were relatively stable, - vaccine 4.5 weeks before this fatal event	Kramer and McClain (1981), Case report
Influenza vaccination	warfarin (CYP2C9)	12	- small but significant increase in the PT ratio before and after vaccination, - maximal increase occurred on day 14 and represented a 7.6% increase over the baseline value	Gray et al. (1983), Non-random
Influenza vaccination	tramadol (CYP2B6 and 3A, bioactivated by CYP2D6)	85-years-old woman and a and 84-years-old man	- hallucinations and other neurologic symptoms six and 5 days after the administration of two different influenza vaccines	Renton et al. (1980), Case report
Influenza vaccination	carbamazepine (CYP1A2 and 2C9, bioactivated by CYP3A)	15-years-old woman	- vaccination 13 days before admission, but it was well tolerated, and no changes were made in her medication, - serum carbamazepine level was 27.5 μg/ml (ataxia and increasing lethargy) at admission and it decreased to 9.1 μg/ml 4 days after admission	Nolin (2008), Case report
Influenza vaccination	phenytoin (CYP2C9 and CYP2C19 substrates and induces CYP2C9, 2C19 and 3 A)	16	- no significant increase in mean serum concentration were observed on days 7 and 14 following the vaccination, - temporary increases of 46–170% mean serum concentration occurred in four subjects	Stenvinkel and Alvestrand (2002), Cohort study
Influenza vaccination	acetaminophen (CYP2E1), alprazolam (CYP3A), antipyrine (CYP1A2, 2B6, 2C8, 2C9, 2C18 and 3A4)	24 (healthy volunteers 9 = acetaminophen, 7 = alprazolam, 8 = antipyrine)	- PK variables were no significantly different (*p* > 0.05) before and 7 and 21 days after vaccination	Nolin et al. (2006), Random

**TABLE 4:** Impact of renal diseases on CYP substrates, explained totally or partially by modulation of CYP activity.

**Inflammation characterized by**	**Victim drugs (CYP concerned)**	**Number of subjects**	**Potential effect of interaction**	**References and design**
Severely impaired renal function vs normal	tolbutamide (CYP2C9)	11 = severe kidney impairment , 7 = normal	- Half-life was prolonged in severely impaired renal function patients (n = 11)	Molanaei et al. (2018), Case-control study
Haemodialyzed patients	alprazolam (CYP3A)	26	- ratio of unconjugated alprazolam to 4-hydroxyalprazolam was correlated with CRP levels (r = 0.49, *p* = 0.01) ADDIN ZOTERO_ITEM CSL_CITATION {"citationID":"Q0Jo8NiX","properties":{"formattedCitation":"(170)","plainCitation":"(170)","dontUpdate":true,"noteIndex":0},"citationItems":[{"id":1099,"uris":["http://zotero.org/users/2161612/items/8PPVMCBX"],"uri":["http://zotero.org/users/2161612/items/8PPVMCBX"],"itemData":{"id":1099,"type":"article-journal","abstract":"OBJECTIVE: To investigate the impact of persistent inflammation in hemodialysis (HD) patients on the pharmacokinetics of alprazolam, a cytochrome P450 (CYP) 3A4 substrate, and its metabolites and the role of HD in the impact of persistent inflammation in this clinical context.\nMETHODS: The study population comprised 26 HD patients (mean age 64 years, range 27-79 years; 19 men, 7 women) who were given 1 mg of alprazolam orally in the evening before the day of HD. Unconjugated and conjugated alprazolam and its 4-hydroxy and α-hydroxy metabolites were measured by liquid chromatography-mass spectrometry at 10, 34 (start of HD) and 38 (end of HD) h after intake. C-reactive protein (CRP) was measured weekly beginning 2 months before study initiation, and alpha 1-acid glycoprotein and 4β-hydroxycholesterol were measured at baseline. CYP3A4 activity was estimated as the ratio of unconjugated alprazolam to 4-hydroxyalprazolam between 10 and 34 h following alprazolam intake.\nRESULTS: After a single dose of alprazolam, plasma concentrations of unconjugated alprazolam and its metabolites decreased gradually, and unconjugated 4-hydroxyalprazolam was eliminated more rapidly than unconjugated alprazolam by HD. In contrast, the plasma concentrations of conjugated alprazolam and its conjugated metabolites increased during the 34 h following drug intake and the subsequent HD decreased their levels by almost 80%. The ratio of unconjugated alprazolam to 4-hydroxyalprazolam was correlated with CRP levels (r(s) = 0.49, P = 0.01). There was no significant correlation between CYP3A4 activity measured by alprazolam (4-hydroxylation) and alpha 1-acid glycoprotein or 4β-hydroxycholesterol. Conjugated alprazolam was also found in the plasma.\nCONCLUSIONS: The correlation between CYP3A4 activity (assessed by alprazolam 4-hydroxylation) and CRP level suggests that inflammation may downregulate CYP3A4 activity. If confirmed, this could have major implications for drug dosing in persistently inflamed patients.","container-title":"European Journal of Clinical Pharmacology","DOI":"10.1007/s00228-011-1163-8","ISSN":"1432-1041","issue":"5","journalAbbreviation":"Eur. J. Clin. Pharmacol.","language":"eng","note":"PMID: 22159869","page":"571-577","source":"PubMed","title":"Metabolism of alprazolam (a marker of CYP3A4) in hemodialysis patients with persistent inflammation","volume":"68","author":[{"family":"Molanaei","given":"Hadi"},{"family":"Stenvinkel","given":"Peter"},{"family":"Qureshi","given":"Abdul Rashid"},{"family":"Carrero","given":"Juan Jesús"},{"family":"Heimbürger","given":"Olof"},{"family":"Lindholm","given":"Bengt"},{"family":"Diczfalusy","given":"Ulf"},{"family":"Odar-Cederlöf","given":"Ingegerd"},{"family":"Bertilsson","given":"Leif"}],"issued":{"date-parts":[["2012",5]]}}}],"schema":"https://github.com/citation-style-language/schema/raw/master/csl-citation.json"}	Molanaei et al. (2012), Cohort study
Haemodialyzed patients	quinine (CYP3A)	44	- significant correlation between the ratio of quinine/3-OH-quinine and median CRP (r = 0.48, *p* = 0.001), orosomucoid (r = 0.44, *p* = 0.003) and IL-6 after 12 h after drug intake (*r* = 0.43, *p* = 0.004), - correlation is no longer significant for IL-6 and orosomucoid after adjustment for age, gender, diabetes mellitus, dialysis vintage, PTH, orosomucoid and medications and it remains borderline for CRP (r = 0.05)	Farrell et al. (1979), Cohort study
End stage renal disease (ESRD) vs. control	warfarin (CYP2C9)	7 = ESRD 6 = control	- 50% (*p* < 0.03) increase plasma warfarin S/R ratio relative to controls	Frye et al. (2006), Case-control study
Moderate and severe kidney impairment vs no/mild kidney impairment	warfarin (CYP2C9)	599 = no/mild 300 = moderate 81 = severe	- patients with moderate kidney impairment required 9.5% lower doses (*p* < 0.001) compared to controls, - patients with severe kidney impairment required 19.1% lower doses (*p* < 0.001) compared to controls, - reduced kidney function was associated with lower dose requirements independently of CYP2C9 and VKORC1 genotype and clinical factors	Grieco et al. (1998), Two cohort studies combined, Case-control study

**TABLE 5:** Impact of liver diseases on CYP substrates, explained totally or partially by modulation of CYP activity.

**Inflammation characterized by**	**Victim drugs (CYP concerned)**	**Number of subjects**	**Potential effect of interaction**	**References and design**
Mild to moderate hepatocellular changes or inactive cirrhosis and severe liver disease vs control	antipyrine (CYP1A2, 2B6, 2C8, 2C9, 2C18 and 3A4)	15 = mild-moderate hepatocellular damage, 13 = inactive cirrhosis, 22 = severe liver disease, 21 = controls	- mean value of hepatic CYP concentration did not differ between patients with mild to moderate hepatocellular changes (less than 50% hepatocytes morphologically abnormal) or inactive cirrhosis and controls and antipyrine half-life did not significantly differ between all groups, - CYP concentration was less in patients with severe liver disease (more than 50% hepatocytes morphologically abnormal or active cirrhosis) and, thus, antipyrine half-life was significantly lower (*p* < 0.01) compared to other groups	Bauer et al. (1994), Case-control study
Liver disease vs. control	caffeine (CYP1A2), mephenytoin (2C19), debrisoquin (2D6), and chlorzoxazone (2E1)	20 = liver disease	- significant decrease in metabolite production in patients with liver disease for CYP2C19 (*p* < 0.001), 2E1 (*p* = 0.0081), 1A2 (*p* = 0.0054) and 2D6 (*p* = 0.0110)	Salmela et al. (1980)
		20 = control	- each probe drug was significantly inversely related to the Pugh score	Case-control study
Chronic active hepatitis and cirrhosis vs. control	antipyrine (CYP1A2, 2B6, 2C8, 2C9, 2C18 and 3A4)	103 = controls, 101 = non-cirrhotic with liver metastases, 102 = chronic active hepatitis, 92 = confirmed cirrhosis, 120 = hepatocellular carcinoma and cirrhosis	- clearance was significantly impaired with respect to healthy volunteers, chronic hepatitis without fibrosis and non-cirrhotic patients with liver metastases, - mean clearance rate of the non-cirrhotic patients with liver metastasis was quite similar to that of patients with healthy livers, - cirrhotic patients with hepatocellular carcinoma also presented significantly impaired clearance compared with that of healthy volunteers and patients with liver metastasis, - elimination of antipyrine may very well be normal in patients with primary or metastatic liver disease, even when there is extensive tumour involvement	Branch et al. (1973), Case-control study
Cirrhotic patient and chronic hepatitis vs. control	antipyrine (CYP1A2, 2B6, 2C8, 2C9, 2C18 and 3A4)	6 = control, 6 = chronic active hepatitis, 5 = cirrhosis	- half-life and clearance were significantly higher and lower respectively in cirrhotic patients compared with healthy subjects, - no significant differences between hepatitis patients and healthy subjects	Schellens et al. (1989), Case-control study
Diabetics with fatty liver, fatty liver with inflammatory changes and with cirrhosis vs diabetics with normal liver	antipyrine (CYP1A2, 2B6, 2C8, 2C9, 2C18 and 3A4)	4 = control, 13 = fatty liver, 33 = fatty liver with inflammation, 6 = cirrhosis	- clearances decreased significantly in diabetics with fatty liver (*n* = 13, *p* < 0.005), in diabetics with fatty liver with inflammatory changes (*n* = 33, *p* < 0.005) and in diabetics with cirrhosis (*n* = 6, *p* < 0.005) as compared to diabetics with normal liver	Teunissen et al. (1984), Case-control study
Cirrhosis vs. normal	tolbutamide (2C9)	10 = cirrhotic patients, 7 = normal	- disappearance rate was reduced in five of ten cases, - half-life was prolonged to 7.8–11.2 h (4.4 h in normal group), - plasma levels after 24 h were 11.4–20.8% of the theoretical initial value (5.3% of the theoretical initial value in normal group)	Molanaei et al. (2018) Case-control study
Acute liver and chronic disease	antipyrine (CYP1A2, 2B6, 2C8, 2C9, 2C18 and 3A4)	14 = control, 38 = liver disease	- half-life was prolonged in patients with liver disease and those with chronic illness had greater increase than those with acute, reversible pathology	Wang et al. (2010), Case-control study
Various liver disease vs. controls	antipyrine (CYP1A2, 2B6, 2C8, 2C9, 2C18 and 3A4), hexobarbital (CYP2C19) and theophylline (CYP1A2)	24 = liver disease, 26 = controls	- clearance of antipyrine, hexobarbital and theophylline are lower than those found in the control subject	Liver disease = Ueda et al. (1963) , Controls = Marino et al. (1998), Case Control
Alcoholic cirrhosis vs. controls	antipyrine (CYP1A2, 2B6, 2C8, 2C9, 2C18 and 3A4)	23 = alcoholic liver cirrhosis, 17 = control	- clearance was significantly lower in patients with alcoholic cirrhosis as compared with healthy volunteers (*p* < 0.001), - the rates antipyrine formations metabolites were not reduced to the same extent	Klotz et al. (1975) Case-control study
Chronic hepatitis	mephenytoin (CYP2C9 and 2C19 and induces 2C9, 2C19 and 3 A)	35 = chronic hepatitis, 153 = controls	- mean metabolite excretion was significantly lower in patients with liver disease (*p* < 0.005)	Laybourn et al. (1986), Case-control study
Liver disease	mephenytoin (CYP2C9 and 2C19 and induces 2C9, 2C19 and 3 A) and debrisoquin (CYP2D6)	18 = liver disease, 8 = controls	- urinary excretion of mephytoin’s metabolite among patients with liver disease was significantly less than among the healthy controls (45% reduction), - the reduction in excretion of mephytoin depended on severity of the disease (28 and 62% decreases for patients with mild and moderate liver disease, respectively), - excretion of debrisoquin’s metabolite was comparable between control and disease groups, as groups with mild or moderate disease	Frye et al. (2002), Case-control study
Cirrhotic vs. control	irbesartan (CYP2C9)	10 = hepatic impairment	- trend for moderate (20–30%) increase in AUC and Cmax values in the cirrhotic group compared with control group but the difference did not meet the predetermined criteria for clinical interest	Toft et al. (1991)
Hepatic impairment vs. control		10 = control	- no significant differences of mean half-life, Cmax, clearance and AUC, - patients with hepatic impairment had higher percentage of cumulative urinary extraction of unchanged irbesartan after multiple dose administration (*p* < 0.05)	Case-control study
Cirrhosis vs. control	meperidine (CYP2B6, 3A4 and 2C19)	10 = cirrhosis, 8 = control	- total plasma clearance was of 664 ± 293 ml/min in cirrhotic patients and of 1′316 ± 383 ml/min in healthy volunteers, - clearance was significantly reduced in cirrhosis patients (*p* < 0.002) ADDIN ZOTERO_ITEM CSL_CITATION {"citationID":"a2nlaknkd00","properties":{"formattedCitation":"(168)","plainCitation":"(168)","dontUpdate":true,"noteIndex":0},"citationItems":[{"id":10553,"uris":["http://zotero.org/users/2161612/items/7HBDUYBB"],"uri":["http://zotero.org/users/2161612/items/7HBDUYBB"],"itemData":{"id":10553,"type":"article-journal","container-title":"Clinical Pharmacology and Therapeutics","DOI":"10.1002/cpt1974164667","ISSN":"0009-9236","issue":"4","journalAbbreviation":"Clin. Pharmacol. Ther.","language":"eng","note":"PMID: 4419525","page":"667-675","source":"PubMed","title":"The effect of cirrhosis on the disposition and elimination of meperidine in man","volume":"16","author":[{"family":"Klotz","given":"U."},{"family":"McHorse","given":"T. S."},{"family":"Wilkinson","given":"G. R."},{"family":"Schenker","given":"S."}],"issued":{"date-parts":[["1974",10]]}}}],"schema":"https://github.com/citation-style-language/schema/raw/master/csl-citation.json"}	Kruger et al. (2009), Case-control study
Cirrhosis vs. control	diazepam (CYP3A)	21 = liver disease (9 alcoholic liver cirrhosis, 8 acute viral hepatitis and 4 chronic active hepatitis), 33 = control	- half-life showed a more than 2-fold prolongation (105.6 ± 15.2 h vs. 46.4 ± 14.2 h, *p* < 0.001) in patients with cirrhosis compared with age-matched control groups, - a decrease in the total plasma clearance of the drug in cirrhosis (*p* < 0.001)	Shelly et al. (1987), Case-control study
Acute viral and chronic active hepatitis vs control			- patients with acute viral hepatitis had a half-life of 74.5 ± 27.5 h and those with active chronic hepatitis of 59.7 ± 23.0 h, as compared to a normal value in this age group of 32.7 ± 8.9 h (*p* < 0.01)	
Cirrhosis and chronic hepatitis B (CHB)	phenacetin (CYP1A2)	106 = cirrhosis, 41 = CHB, 82 = controls	- clearance decreased by 91.2% (*p* < 0.01) and 67.7% (*p* < 0.005) in the patients with cirrhosis (*n* = 106) and chronic hepatitis B (*n* = 41), respectively	Schoergenhofer et al. (2018), Case-control study

**TABLE 6:** Impact of lung diseases on CYP activities.

**Inflammation characterized by**	**Victim drugs (CYPs concerned)**	**Number of subjects**	**Potential effect of interaction**	**References and design**
COPD exacerbation	clozapine (CYP1A2)	52-year-old woman	- symptoms of clozapine toxicity, - serum levels = 1400 ng/ml (References = 350–700 ng/ml)	Luong et al. (2016), Case reports
Chronic obstructive lung (COLD) and pulmonary disease caused by α1-antitrypsin (AAT) deficiency vs. control	antipyrine (CYP1A2, 2B6, 2C8, 2C9, 2C18 and 3A4)	35 = AAT, 25 = COLD, 31 = control	- clearance was not different in AAT and COLD patients (*p* > 0.2), - clearance significantly higher in healthy volunteers than in patients with COLD (18%, *p* < 0.01)	Bilbao-Meseguer et al. (2018), Case-control study

**TABLE 7:** Impact of cardiac diseases on CYP substrates, explained totally or partially by modulation of CYP activity.

**Inflammation characterized by**	**Victim drugs (CYPs concerned)**	**Number of subjects**	**Potential effect of interaction**	**References and design**
Congestive heart failure	caffeine (CYP1A2), mephenytoin (2C19), dextromethorphan (2D6), chlorzoxazone (2E1)	16	- IL-6 levels were inversely correlated to CYP1A2 (r = -0.56, *p* = 0.0235) and CYP2C19 (r = -0.63, *p* = 0.0094) activities, - TNF-α was inversely correlated to CYP2C19 (*r* = −0.61, *p* = 0.0118) activity, - no significant relationship between IL-6 and TNF-α with CYP2D6 and 2E1 activities	Pirttiaho et al. (1984), Cohort study

**TABLE 8:** Impact of critically ill patients on CYP substrates, explained totally or partially by modulation of CYP activity.

**Inflammation characterized by**	**Victim drugs (CYPs concerned)**	**Number of subjects**	**Potential effect of interaction**	**References and design**
Septicaemia with shock and respiratory failure and multiple organ failure (two or more organ dysfunction)	theophylline (CYP1A2) and ethylene-diamine (CYP3A)	6	- 10–66% reduction of theophylline clearance as compared to healthy volunteers. Half-life was 18.8 h compared to a normal value of 6 h, - 54% reduction of ethylenediamine clearance and half-life was 2.3 h, which is 5 times the normal value of 0.55 h	Zysset and Wietholtz (1988), Cohort study
Critically ill patients (ICU) with sepsis	atorvastatin (CYP3A)	12 = ICU with sepsis	- 18-fold higher Cmax (*p* < 0.001) and 15-fold higher AUC (*p* < 0,01)	Gravel et al. (2019)
vs control		5 = healthy volunteers		Case-control study
Critically ill patients	midazolam (CYP3A)	6	- CYP3A downregulation is proportional to the severity of the patient’s illness and reversible, - normal values from other studies ADDIN ZOTERO_ITEM CSL_CITATION {"citationID":"a2lr6jrcbos","properties":{"formattedCitation":"(139)","plainCitation":"(139)","noteIndex":0},"citationItems":[{"id":10589,"uris":["http://zotero.org/users/2161612/items/8UL6EWVY"],"uri":["http://zotero.org/users/2161612/items/8UL6EWVY"],"itemData":{"id":10589,"type":"article-journal","container-title":"The Journal of Pharmacy and Pharmacology","DOI":"10.1111/j.2042-7158.1983.tb02960.x","ISSN":"0022-3573","issue":"6","journalAbbreviation":"J. Pharm. Pharmacol.","language":"eng","note":"PMID: 6135777","page":"378-382","source":"PubMed","title":"Comparative plasma pharmacokinetics of theophylline and ethylenediamine after the administration of aminophylline to man","volume":"35","author":[{"family":"Cotgreave","given":"I. A."},{"family":"Caldwell","given":"J."}],"issued":{"date-parts":[["1983",6]]}}}],"schema":"https://github.com/citation-style-language/schema/raw/master/csl-citation.json"} (139)	Preston et al. (2001), Case-control study
Multiply injured patients vs. healthy volunteers	mephenytoin (CYP2C19), chlorzoxazone (CYP2E1), dapsone (multiple CYP) and flurbiprofen (CYP2C9)	23 = multiple injured patients, 90 = control	- CYP2C19 and 2E1 activity significantly reduced in trauma patients as compared to healthy volunteers, - CYP2C9 and multiple CYP activities (dapsone) higher after injury as compared to healthy volunteers, - CYP2C19 and 2E1 activities correlated with MODS and MOF scores	Marques et al. (2002), Case-control study
Critically ill patients	clopidogrel (bioactivated by CYP2C19), pantoprazole (CYP2C19)	43 = clopidogrel, 16 = pantoprazole	- median ratio of clopidogrel active metabolite to clopidogrel concentration was 0.6 and this ratio was 48-fold higher (*p* < 0.001) in healthy volunteers, - 70% of critically ill patients were insufficiently treated with clopidogrel, - 5-fold increased pantoprazole half-life	Akhlaghi et al. (2012), Cohort study

**TABLE 9:** Impact of diabetes on CYP substrates, explained totally or partially by modulation of CYP activity.

**Inflammation characterized by**	**Victim drugs (CYPs concerned)**	**Number of subjects**	**Potential effect of interaction**	**References and design**
Non-insulin dependent (NID) diabetic subjects with fatty liver vs. healthy subjects	antipyrine (CYP1A2, 2B6, 2C8, 2C9, 2C18 and 3A4)	21 = diabetes, 11 = control	- NID diabetic subjects with fatty liver have lowered hepatic drug metabolizing enzyme capacity as assessed per unit weight of liver tissue compared with healthy subjects (*p* < 0.01), - the relative clearance rate was significantly slower and the hepatic CYPs concentration lower than in non-diabetic controls (*p* < 0.01)	Wadhawan et al. (2000), Case-control study
Diabetes patients with normal liver	antipyrine (CYP1A2, 2B6, 2C8, 2C9, 2C18 and 3A4)	4 = diabetes, 13 = controls	clearance decrease significantly (*p* < 0.005) between diabetes patients with normal liver compared to controls	Teunissen et al. (1984), Case-control study
Type I and type II diabetes vs. controls	antipyrine (CYP1A2, 2B6, 2C8, 2C9, 2C18 and 3A4)	30 = diabetes (15 T1D and 15 T2D), 21 = controls (12 for T1D and 9 for T2D)	- half-life was reduced by 44% compared to the controls (*p* = 0.002), whereas the resulting plasma clearance did not differ between controls and type I diabetics (T1D), - Type II diabetics (T2D) showed a 31% increase in plasma half-life (*p* = 0.05) and they had a significant decrease in corresponding clearance (*p* = 0.02)	Darakjian et al. (2021), Case-control study
Type I and type II diabetes vs. controls	antipyrine (CYP1A2, 2B6, 2C8, 2C9, 2C18 and 3A4), caffeine (CYP1A2) and dextromethorphan (CYP2D6)	15 = T1D, 16 = T2D, 16 = controls	- metabolism was significantly higher in T1D patients than in the patients with T2D and in healthy volunteers, - no change in metabolism between T2D and controls, - CYP1A2 activity was 34 and 42% higher in patients with T1D compared with controls and patients with T2D respectively but these changes did not reach the statistical significance (*p* = 0.11), - no change between groups concerning the CYP2D6 phenotype distribution	Matzke et al. (2000), Case-control study
Type II diabetes vs control	caffeine (CYP1A2) bupropion (CYP2B6), tolbutamide (CYP2C9), omeprazole (CYP2C19), dextrometorphan (CYP2D6), chlorzoxazone (CYP2E1) and CYP3A (midazolam)	38 = T2D, 35 = control	CYP2B6, CYP2C19 and CYP3A activities were decreased by about 45% (*p* = 0.01), 46% (*p* = 0.001) and 38% (*p* < 0.0001) respectively in T2D patients and multivariate models showed that IFN-γ and TNF-α, pro-inflammatory cytokines, partly explain these variations, - CYP1A2 and CYP2C9 metabolic activity were increased in T2D patients (*p* = 0.008 and *p* = 0.0008, respectively) at first sight but this is no longer significant when they have been adjusted for age and gender (*p* = 0.07 and *p* = 0.05, respectively), - CYP2D6 and CYP2E1 activities were not affected by diabetic status (*p* = 0.75 and *p* = 0.78, respectively), - phenotypes were extrapolated from genotypes because patients did not take other co-medications and there is no interaction between genotype/phenotype classification and diabetic status	Lucas et al. (1998), Case-control study
Type II diabetes vs. control	caffeine (CYP1A2)	57 = T2D, 146 = control	- metabolic activity of CYP1A2 was significantly increased in T2D patients compared to control (*p* = 0.010), - but when the 19 diabetic patients who are under insulin injection were removed, the difference was no longer significant (*p* = 0.121)	Dyer et al. (1994), Case-control study
Insulin dependent (ID) diabetes patients vs. control	caffeine (CYP1A2) and debrisoquin (CYP2D6)	28 = ID diabetes patients, 22 = healthy volunteers	- no significant differences for CYP2D6 activity and a significant increase in CYP1A2 activity in diabetes patients (*p* < 0.0001)	Wang et al. (2003), Case-control study
T1D and T2D vs. control	caffeine (CYP1A2)	10 = T1D; 8 = controls, 9 = T2D; 9 = controls	the apparent volume of distribution, apparent clearance, half-life, and peak concentrations of caffeine did not differ between both type of diabetes and controls	Sotaniemi et al. (2002), Case-control study
Diabetic patients vs. controls	tolbutamide (CYP2C9)	10 = diabetic patients, 7 = control	half-life in diabetic patients revealed no significant difference with normal subjects ADDIN ZOTERO_ITEM CSL_CITATION {"citationID":"yU0UBeFO","properties":{"formattedCitation":"(115)","plainCitation":"(115)","noteIndex":0},"citationItems":[{"id":10235,"uris":["http://zotero.org/users/2161612/items/ELGVD5C6"],"uri":["http://zotero.org/users/2161612/items/ELGVD5C6"],"itemData":{"id":10235,"type":"article-journal","container-title":"Diabetes","DOI":"10.2337/diab.12.5.414","ISSN":"0012-1797","journalAbbreviation":"Diabetes","language":"eng","note":"PMID: 14067739","page":"414-419","source":"PubMed","title":"DISAPPEARANCE RATE OF TOLBUTAMIDE IN NORMAL SUBJECTS AND IN DIABETES MELLITUS, LIVER CIRRHOSIS, AND RENAL DISEASE","volume":"12","author":[{"family":"Ueda","given":"H."},{"family":"Sakurai","given":"T."},{"family":"Ota","given":"M."},{"family":"Nakajima","given":"A."},{"family":"Kamii","given":"K."},{"family":"Maezawa","given":"H."}],"issued":{"date-parts":[["1963",10]]}}}],"schema":"https://github.com/citation-style-language/schema/raw/master/csl-citation.json"} (115)	Molanaei et al. (2018), Case-control study
Diabetes mellitus vs. controls	paracetamol (CYP2E1)	19 = diabetes mellitus, 10 = healthy volunteers	- half-life was significantly increased (*p* < 0.001) with a corresponding decrease in clearance (*p* < 0.001) when compared with healthy volunteers, - clearance in patients with T2D was significantly decreased compared to T1D patients (*p* < 0.01) but it was not the case for its half-life, - the distribution volume was increased in patients with T1D compared to patients with T2D (*p* > 0.05)	Korrapati et al. (1995), Case-control study
Type II diabetes vs control	amlodipine (CYP3A)	18 = T2D, 20 = control	-no significant difference in AUC in hypertensive patients with and without T2D	Bechtel et al. (1988), Case-control study
Type II diabetes vs control	nisoldipine (CYP3A) and lidocaine (CYP3A)	17 = T2D, 10 = control	- the apparent clearances of both nisoldipine enantiomers in the hypertensive patients with T2D are significantly lower than in hypertensive control patients (*p* < 0.05), - higher ratio of plasma lidocaine/MEGX concentration for diabetic group than in control group (*p* < 0.05), - means that CYP3A4 activities were decreased in the diabetic groups, - significant correlations were found (*p* < 0.05) between the MR of lidocaine and the apparent clearance of nisoldipine enantiomers obtained for both groups	Urry et al. (2016), Case-control study
Diabetes vs. control	CyA (CYP3A)	7 = diabetes, 10 = control	-No difference was found in daily dose needed between both groups (*p* = 0.55) but metabolite-parent concentration ratios for all metabolites except one (AM4N, *p* = 0.93) were significantly lower in diabetic patients (0.0001 < *p*-value < 0.04)	Idle et al. (1978), Case-control study
Diabetes vs. control	CyA (CYP3A)	8 = diabetes, 9 = control	AUC adjusted with dosage was significantly lower in diabetic group (*p* = 0.03) ADDIN ZOTERO_ITEM CSL_CITATION {"citationID":"atdeho0nge","properties":{"formattedCitation":"(194)","plainCitation":"(194)","dontUpdate":true,"noteIndex":0},"citationItems":[{"id":11162,"uris":["http://zotero.org/users/2161612/items/KYQT5CPG"],"uri":["http://zotero.org/users/2161612/items/KYQT5CPG"],"itemData":{"id":11162,"type":"article-journal","abstract":"BACKGROUND AND OBJECTIVES: Long-term diabetes mellitus may affect the absorption, distribution and metabolism of immunosuppressive agents used after organ transplantation. The aims of this study were to characterize ciclosporin pharmacokinetics in blood and plasma and to compare the ciclosporin unbound concentration and the blood : plasma concentration (B : P) ratio in diabetic kidney transplant recipients.\nPATIENTS AND METHODS: Ciclosporin 12-hour steady-state pharmacokinetics were studied in eight diabetic and nine nondiabetic patients. Ciclosporin concentrations in whole blood and in plasma were measured using liquid chromatography-tandem mass spectrometry, and the ciclosporin fraction unbound (f(u)) was determined by an equilibrium dialysis method utilizing [(3)H]ciclosporin as a tracer. Oral absorption of paracetamol (acetaminophen) was used as a marker for gastric emptying.\nRESULTS: In diabetic patients, the time to the peak blood ciclosporin concentration at steady state (t(max)(,ss)) was prolonged (128 minutes vs 93 minutes in nondiabetic patients, p < 0.01) and, on average, the paracetamol t(max) was prolonged by 30 minutes. The whole-blood dose-normalized area under the concentration-time curve from 0 to 12 hours (AUC(12)) was marginally lower in diabetic patients (p = 0.09) and the plasma AUC(12) was significantly lower (p = 0.03). The ciclosporin f(u) was numerically higher in diabetic patients (1.20 +/- 0.65% vs 0.72 +/- 0.28% in nondiabetic patients, p = 0.066); however, the unbound concentration values were essentially similar in the two groups (0.58 +/- 0.76 microg/L in diabetic patients and 0.52 +/- 0.48 microg/L in nondiabetic patients; p = 0.59). No difference was observed in the ciclosporin B : P ratio between the two groups.\nCONCLUSION: This study indicates that diabetes delays ciclosporin absorption, reduces ciclosporin exposure and increases the ciclosporin f(u) but not the pharmacologically active unbound concentration.","container-title":"Clinical Pharmacokinetics","DOI":"10.2165/00003088-200847110-00004","ISSN":"0312-5963","issue":"11","journalAbbreviation":"Clin Pharmacokinet","language":"eng","note":"PMID: 18840028","page":"733-742","source":"PubMed","title":"Blood and plasma pharmacokinetics of ciclosporin in diabetic kidney transplant recipients","volume":"47","author":[{"family":"Mendonza","given":"Anisha E."},{"family":"Gohh","given":"Reginald Y."},{"family":"Akhlaghi","given":"Fatemeh"}],"issued":{"date-parts":[["2008"]]}}}],"schema":"https://github.com/citation-style-language/schema/raw/master/csl-citation.json"}	Baer et al. (1986), Case-control study
Diabetes vs. control	CyA (CYP3A)	36 = diabetes, 67 = control	- no difference was found concerning dose and through levels	Smolen et al. (2016), Case-control study
Type I and II diabetes vs control	chlorzoxazone (CYP2E1)	7 = T1D, 15 = T2D, 42 = controls	- no difference was found concerning CYP2E1 activity between groups	Mayo et al. (2000), Case-control study
Type II diabetes vs. control	quinine (CYP3A)	12 = T2D, 10 = controls	- PK parameters were comparable in the two groups (*p* > 0.02)	Daneshtalab et al. (2006), Case control study
Type I and II diabetes vs control	chlorzoxazone (CYP2E1)	14 = T1D, 8 = T2D, 10 = controls	- 2-fold increase in the oral clearance (*p* < 0.05) in T2D patients compared with T1D and controls, - no difference in oral clearance between T1D and controls	Tracy et al. (1999), Case-control study
Type I and type II diabetes	antipyrine (CYP1A2, 2B6, 2C8, 2C9, 2C18 and 3A4)	139 = T1D (120 = controls), 99 = T2D (70 = controls)	- clearance decreased in T2D patients as compared to controls, - metabolism is rapid in T1D patients	Goktaş et al. (2015), Case-control study
Type 1 diabetes vs controls	theophylline (CYP1A2)	8 = T1D, 8 = controls	- mean plasma clearance and elimination half-life did not differ significantly between the 2 groups	Sanaee et al. (2011), Case-control study
Gestational diabetes vs. pregnant women	metoprolol (CYP2D6)	10 = diabetes, 13 = control	- PK of the metoprolol isomers in the pregnant women and in gestational diabetes groups did not differ significantly, except for the R-metoprolol half-life (*p* < 0.05)	Schneider et al. (1976), Case-control study
Gestational diabetes vs. pregnant women	lidocaine (CYP3A)	6 = diabetes, 10 = control	- the ratios of lidocaine and its metabolite MEGX concentrations (lidocaine/MEGX ratio) at 15 and 30 min were significantly higher in the pregnant women with gestational diabetes mellitus compared to the normal pregnant women (58.34 vs. 23.21 at 15 min and 37.52 vs. 15.80 at 30 in, *p* < 0.05)	Lebwohl et al. (2018), Case-control study

**TABLE 10:** Impact of autoimmune diseases on CYP substrates, explained totally or partially by modulation of CYP activity.

**Inflammation characterized by**	**Victim drugs (CYPs concerned)**	**Number of subjects**	**Potential effect of interaction**	**References and design**
Psoriasis vs healthy volunteers	venlafaxine (CYP2D6)	13 = psoriasis, 11 = control	- PK of the enantiomers and of its metabolites were not altered as compared to control	Lang et al. (1996) Case-control study
Systemic lupus erythematosus (SLE) vs. healthy controls	debrisoquin (CYP2D6)	42 = SLE, 147 = control	- In patients with SLE, there is an inhibition in the metabolism of debrisoquin compared to controls because there is significantly more PM patients in patients group (*p* < 0.04)	Tidball (2005), Case-control study
Proctitis vs healthy volunteers	/	11	- patients who suffered from proctitis showed a lower CYP2E1 and 3A4 gene expression in rectal mucosa with severe inflammation compared to normal mucosa (*p* < 0.05), - no significant difference for CYP3A5 (*p* = 0.08)	Baigrie et al. (1992), Cohort study
Behçet’s disease vs. healthy subjects	losartan (CYP2C9)	52 = Behçet’s disease, 73 = control	- the MR (losartan/E-3174) significantly increase (*p* = 0.002) compare to controls already included who genetic variants and losartan oxidation were already known, - in patients with the wild type CYP2C9 genotype (*1/*1), the MR significantly increased in patients with Behçet’s disease compared to controls (*p* = 0.006) but there is no significant differences found for other CYP2C9 genotype	Bergin et al. (2011), Case-control study
Rheumatoid arthritis (RA) vs. healthy volunteers	verapamil (CYP3A4, 1A2, 2C8, 2C9 and 2C18)	8 = RA, 8 = controls	- less metabolized and bound to protein in patients with RA compared to controls, - AUC of verapamil and norverapamil were significantly higher in patients with RA as compared to controls thus, there is no changes in metabolite to parent drug ratio	Haas et al. (2003), Case-control study
Active and controlled rheumatoid arthritis vs healthy subjects	losartan (CYP2C9)	14 = active RA, 12 = controlled RA, 12 = controls	- PK not significantly altered but AUC of its pharmacologically active metabolite was significantly decreased , - MR exhibited a significant correlation with disease severity (*r* = −0,35, *p* < 0.05)	Lenoir et al. (2020), Case-control study
Rheumatoid arthritis	/	49 = RA	- cytokines such as TNF-α, IL-1β and IL-17 increase the CYP7B activity in synovial tissue, - TGF-β down-regulate the CYP7B activity and it results in enhanced formation of 7α-OH-DHEA in the arthritic joint, which may contribute to the maintenance of the inflammation and, thus, the chronicity of the inflammation response	Mostowik et al. (2015), Cohort study
active Crohn’s disease (CD), Crohn’s disease in remission and healthy subjects	verapamil (CYP3A4, 1A2, 2C8, 2C9 and 2C18)	22 = CD remission, 14 = CD active, 9 = controls	- plasma S-verapamil concentration in patients with active CD was significantly higher than in both healthy controls and patients in CD remission (*p* < 0.001) but not between healthy controls and Crohn’s disease remission, - same tendency was seen for R-verapamil but there is no statistical significance, - as in RA patients, the ratio AUC of both S and R norverapamil over their corresponding verapamil enantiomers were not significantly different among the 3 groups of subjects, - there was no higher PD response in patients due to higher verapamil level	Bernlochner et al. (2010), Case-control study
Crohn’s disease vs. control	propranolol (CYP2D6)	10 = Crohn’s disease, 12 = healthy subjects	- levels were significantly higher in the 10 patients with Crohn’s disease than those of the controls (*p* < 0.05)	Harvey and Morgan (2014), Case-control study
Celiac disease	/	9	- reduction in the intestinal content of CYP3A in patients with celiac disease before treatment with a gluten-free diet and increase in intestinal CYP3A protein after the diet	Kacevska et al. (2008), Cohort study

**TABLE 11:** Impact of surgery on CYP substrates, explained totally or partially by modulation of CYP activity.

Inflammation characterized by	Victim drugs (CYPs concerned)	Number of subjects	Potential effect of interaction	References and design
Surgery	clozapine (CYP1A2)	49-year-old man	- clozapine and norclozapine levels were 1130 ng/dl and 297 ng/dl, respectively (ratio 3.8:1), 4 days after surgery. On day 2, dosage was reduced due to persistent sedation	Luong et al. (2016), Case reports
(a) Surgery	/	16 (5 a, 6 b and 5 c)	- ERMBT results significantly declined in all groups compared with before surgery	Chen et al. (1994)
abdominal aortic bypass graft	carbon-14 [^14^C] ERMBT (CYP3A)		- a trend toward difference in ERMBT results between surgery but didn’t reach statistical significance (*p* = 0.06)	Cohort study
colon resection			- the nadir ERMBT result was significantly and negatively correlated (*r* = -0.541, *p* = 0.03) with peak IL-6 concentration	
peripheral vascular bypass graft			- test results were significantly different if patients IL-6 peak concentration was IL-6 > 100 pg/ml or <100 pg/ml (35.5 vs. 74.7%, *p* < 0.001)	
Hip surgery	caffeine (CYP1A2), bupropion (CYP2B6), flurbiprofen (CYP2C9), omeprazole (CYP2C19), dextromethorphan (CYP2D6) and midazolam (CYP3A)	30	- CYP2C19 and 3A MR decreased by 57% (*p* = 0.0002) and 61% (*p* ≤ 0.0001) respectively with the nadir at D3, - CYP1A2 MR decreased by 53% (*p* ≤ 0.0001) with the nadir at D1, - CYP2B6 and 2C9 MR increased by 120% (*p* < 0.0001) and 79% (*p* = 0.0018), respectively and peaked at d1, - No change in CYP2D6 MR	Rivory et al. (2002), Cohort study
percutaneous coronary intervention	clopidogrel (bioactivated by CYP2C19)	50	- prolonged post-angioplasty increase is associated with lower platelets’ response to clopidogrel	Alexandre et al. (2007), Cohort study
percutaneous coronary intervention	clopidogrel (bioactivated by CYP2C19)	1′223	- platelet aggregation was significantly higher in patients with elevated CRP levels compared to patients with normal CRP levels (*p* < 0.001)	Charles et al. (2006), Cohort study

**TABLE 12:** Impact of cancer on CYP substrates, explained totally or partially by modulation of CYP activity.

**Inflammation characterized by**	**Victim drugs (CYPs concerned)**	**Number of subjects**	**Potential effect of interaction**	**References and design**
Liver metastasis before cytostatic treatment vs. healthy controls	antipyrine (CYP1A2, 2B6, 2C8, 2C9, 2C18 and 3A4)	12 = liver metastasis, 12 = controls	- no significant difference between patients with liver metastases before cytostatic treatment and controls	Williams et al. (2000), Case-control study
Bone marrow transplantation for haematological malignancies (radiation and chemotherapy)	CyA (CYP3A)	6	- concentration peak value occurred 15.8 days after bone marrow transplantation and it’s corresponded to a 3- or 4-fold increase relative to the steady state day (*p* > 0.015), - CyA concentration peak and IL-6 peak levels are interdependent because there was a correlation between these two parameters (*r* = 0.794, *p* = 0.03)	Burns et al. (2014), Cohort study
Cancer	ERMBT (CYP3A)	40	- patients with CRP >10 mg/L had an average 30% reduction in CYP3A4 metabolic activity (*p* = 0.0062), - 1/Tmax values were negatively correlated with both CRP (*r* = −0.64, *p* < 0.00001) and α-glycoprotein (*r* = -0.45, *p* < 0.005), - 3 patients were treated by a CYP3A4 inhibitor while 4 patients were on long-term treatment with dexamethasone (inducer) but correlation with CRP remained significant (r = −0.55, *p* = 0.002) after removal of these patients	Helsby et al. (2008), Cohort study
Advanced cancer patients with normal liver function	midazolam and docetaxel (CYP3A)	56	- high midazolam concentration and free docetaxel AUC were associated with sever neutropenia (and conversion to febrile neutropenia), - high midazolam concentration was correlated with elevated ferritin level (r = 0.32, *p* = 0.02) (indicator of an inflammatory state), - according to authors, inflammation favors a reduction in CYP3A activity and thus, could lead to an overexposure to its substrates	Yasu et al. (2017), Cohort study
Advanced cancer patients who were suitable for palliative chemotherapy	docetaxel (CYP3A)	68	- occurrence of grade 3/4 non-haematological toxicities were not associated with high docetaxel exposure but with baseline concentrations of AAGP (*p* = 0.03) and CRP (*p* = 0.05), - results from correlation analysis between inflammation markers and docetaxel clearance were not given, as the results from EBT	Mafuru et al. (2019), Non-randomized clinical trial
Cancer patients vs healthy subjects	omeprazole (CYP2C19)	16 = cancer, 77 = controls	CYP2C19 activity differed significantly (*p* < 0.0001) in the EM cancer patients compared of the References population with EM genotype	Piscitelli et al. (1998), Case-control study
Multiple myeloma	proguanil (CYP2C19)	25	- significant discordance between the CYP2C19 activity predicted by genotype and the measured phenotype (*p* < 0.0001), - no significant difference in CRP and IL-6 concentrations between discordant and concordant subjects (*p* = 0.072 and *p* = 0.694, respectively)	Elkahwaji et al. (1999), Cohort study
Advanced cancer	omeprazole (CYP2C19)	31	- comparison of the predicted phenotype from genotype and the measured MR of CYP2C19 found a statistically discordance (*p* < 0.0005), - of the 30 cancer patients with genotypic EM status, 11 were CYP2C19 PM, - no significant correlation between the levels of any individual cytokine (CRP, IL-1β, Il-1α, IL-6, TNF-α, TGF-β and CRP) and CYP2C19 metabolic activity	Israel et al. (1993), Cohort study
Hematopoietic cell transplantation	voriconazole (CYP3A4 and CYP2C19)	67	- CRP levels were significantly correlated (*r* = 0.22, *p* < 0.001), - higher voriconazole trough concentration >1.0 ug/ml was observed in higher CRP level >4 mg/dl	Jonkman et al. (1989), Cohort study
Hematologic patients	voriconazole (CYP3A4 and CYP2C19)	113	- concentration was significantly correlated with IL-18 in acute myeloid (*r* = 0.456, *p* < 0.0001), acute lymphoblastic (*r* = 0.317, *p* = 0.019), and chronic myeloid leukaemia (*r* = 0.737, *p* = 0.04), - concentration and TGF-β1 were correlated (r = 0.436, *p* < 0.001) in acute myeloid leukaemia patients only, - according to authors, IL-6 level could partially predict the voriconazole trough concentration because these two factors were weakly inversely correlated in hematologic patients regardless of underlying disease	Williams et al. (1987), Cohort study
Hepatocellular carcinoma	phenacetin (CYP1A2)	148 = carcinoma, 82 = controls	- clearance did not significantly differ between the healthy participants and patients with hepatocellular carcinoma	Schoergenhofer et al. (2018), Case-control study

**TABLE 13:** Impact of therapies with immunomodulator on CYP substrates, explained totally or partially by modulation of CYP activity.

**Inflammation characterized by**	**Victim drugs (CYPs concerned)**	**Number of subjects**	**Potential effect of interaction**	**References and design**
Treatment with IL-2	indinavir (CYP3A)	8 = HIV seropositive patients (observational), 9 = HIV seropositive patients (prospective)	- in the HIV seropositive-patients, the mean concentration of indinavir was significantly increased on day 5 of IL-2 therapy, - in the nine HIV seropositive-patients, the mean indinavir AUC increased significantly by 88% between day 1 and day 5 of IL-2, - mean IL-6 concentrations during IL-2 therapy increased between day1 and day5 from 4- to 86-fold, - study combines observations made in one observational and one prospective (as part of a phase II trial) studies	Williams and Farrell (1986), Cohort study and non-randomized
Treatment with IL-2	/	5 = 3 or 6x10^6^/m^2^ units of IL-2, 6 = 9 or 12x10^6^/m^2^ units of IL-2, 7 = 0 units of IL-2, Patients with cancer	- in non-tumorous liver fragment removed with the tumor in each patients, authors observed that CYPs proteins (CYP1A2, 2C, 2E1 and 3A), monooxygenase activities of methoxyresorufin and erythromycin and total CYPs were significantly decreased only in the group of patients treated with highest doses of IL-2, compared to control	Furlanut et al. (2010), Randomized clinical trial
Treatment with IFN-α	theophylline (CYP1A2), antipyrine (CYP1A2, 2B6, 2C8, 2C9, 2C18 and 3A), hexobarbitone (CYP2C19)	7	- no significant difference in TNF-α, IL-1β, IL-6 and CRP activities after both acute (initiation) and chronic (2 weeks) IFN-α injections compared to baseline, except for TNF-α activity that significantly decreased after chronic therapy, - significant effects of acute IFN-α administration on the oral clearance of the three probe drugs were not detected, - chronic exposure to IFN-α was associated with a significant lowering clearance (33% compared with baseline, *p* < 0.05) but no significant correlations were observed between the changes in theophylline clearance and changes in serum cytokines or acute phase proteins, - chronic IFN-α therapy decreased antipyrine oral clearances by 20% but this did not reach statistical significance and it appeared to have no effect on the metabolism of racemic hexobarbitone	Sulkowski et al. (2005), Cohort study
Treatment with IFN-α	aminophylline (CYP1A2)	12 = healthy volunteers	- after IFN-α treatment in healthy volunteers, there were significant 10–15% increases (*p* < 0.05) in the terminal elimination half-life and AUC of aminophylline administered intravenously, - the total clearance showed a comparable decrease (*p* < 0.05)	Gupta et al. (2007), Non-randomized
Treatment with IFN	theophylline (CYP1A2)	5 = hepatitis B, 4 = healthy subjects	- a reduction of theophylline elimination was observed in 8 subjects (remaining subject was a healthy control) and was ranged from 33 to 81%, compared to initial theophylline clearance study, - no impact of the hepatitis on these results because there was no clinical or biochemical change in the liver disease, - a second theophylline clearance study was done 4 weeks after the interferon’s injection and it was back to initial value	Hellman et al. (2003), Non-randomized
Treatment with IFN-α	antipyrine (CYP1A2, 2B6, 2C8, 2C9, 2C18 and 3A)	5 = hepatitis B, 4 = healthy subjects	recombinant leukocyte α-interferon reduced the antipyrine clearance by 16% (*p* < 0.01) and the half-life increased but this was not significant	Brennan et al. (2013), Non-randomized
Treatment with IFN-α	warfarin (CYP2C9)	52 year-old-woman	- her prothrombin time increased to 16.7–20.4 s with a rise in serum warfarin concentration from <0.8 μg/ml to 5.2 μg/ml 10 days after the onset of IFN-α therapy, - dose was reduced and both anticoagulation and serum warfarin concentration had returned to nearly baseline values	Adachi et al. (1995), Case report
Treatment with IFN-α-2b	acenocoumarol (CYP2C9)	46-year-old-woman	- at the beginning of the treatment, anticoagulant effect of acenocoumarol increased (thrombotest decreased from 30–35–19%), - when IFN-α-2b dosage decreased because of infection remission, anticoagulant effect decreased (thrombotest increased from 25–40–69%), - it led to the adaptation of the dosage of acenocoumarol to be on thrombotest range, - anticoagulation level decreased from 1 day after injection to 2 or 3 days later	Serratrice et al. (1998), Case report
Treatment with IFN-α-2b	ERMBT (CYP3A)	6 = chronic hepatitis C, 4 = healthy controls	- ERMBT before and 20–26 h after IFN-α-2b injection, - IFN-α-2b induced a small significant decrease in ERMBT (*p* < 0.05), - at baseline CYP3A4 activity was lower in patients with hepatitis C but the effect of IFN appeared to be not different	Craig et al. (1993), Non-randomized
Treatment with IFN-α	cyclophosphamide (CP) (CYP2B6 active metabolite and CYP2C9, 2C19 and 3A substrate)	10	- administration of IFN-α before CP caused a 63% decrease in its clearance (*p* = 0.004) compared to an administration of IFN-α 24 h after CP, - there is a 45% decrease in exposure of CP active metabolite’s (4-OHCP) when IFN-α was administered before CP, expressed as AUC (*p* = 0.002), compared with that observed when IFN-α was administered 24H after CP, - this resulting in a greater decrease in leukocyte count (45%, *p* = 0.02) when IFN-α was given after CP in the 10 patients with multiple myeloma	Hassan et al. (1999), RCT
Treatment with IFN-α-ribavirin	dextromethorphan (CYP3A4 and CYP2D6, by measuring different metabolite) and caffeine (CYP1A2)	14	- mean CYP3A4 activity increased from 0.18 ± 0.06 in patient with HCV before beginning of IFN-α-ribavirin treatment to 0.48 ± 0.53 1 month after but this did not reach statistical significance (*p* = 0.19) - a similar evolution of CYP2D6 activity could be observed during the first month of treatment (148 ± 0139 to 421 ± 641, *p* = 0.08), - CYP1A2 activity did not changed, going from 0.39 ± 0.11 before treatment to 0.32 ± 0.13 after 1 month, - pretreatment CYP3A4 and CYP2D6 activities of the 14 studied patients were significantly lower than those observed in 35 healthy volunteers (*p* = 0.0006 and *p* = 0.0008 respectively), - after 1 month of antiviral treatment, CYP3A4 and 2D6 did not differ significantly from those in healthy volunteers, probably because of the recovery of HCV patients	Becquemont et al. (2002), Non-randomized
Treatment with IFN-α-2b	caffeine (CYP1A2), mephenytoin (CYP2C19), debrisoquin (CYP2D6), chlorzoxazone (CYP2E1) and dapsone (CYP2C8 and CYP2C9)	17 = patients with high-risk resected melanoma	- IFN-α-2b inhibits immediately the activity of CYP1A2 (*p* = 0.001) and 2D6 (*p* < 0.001) in patients with high-risk resected melanoma, - inhibition of CYP2C19 was detected for the first time at day 26 (*p* < 0.001) after the initiation of high-dose IFNα-2b treatment (20 MU/m2/day i.v for 5 days/weeks during 4 weeks and 10 U/m2/day s.c for 3 days/week x 48 weeks), - no significant inhibition was seen for CYP2E1	Islam et al. (2002), Cohort study
Treatment with peginterferon-α-2b	dextromethorphan (CYP2D6) and, fluoxetine (CYP2D6 active metabolite)	20	- MR before and after initiation of peginterferon-α-2b and ribavirin therapy go from 0.10 ± 0.40 to 0.04 ± 0.09 and that’s mean that metabolite production of dextromethorphan increased after hepatitis C, but it is not significant (*p* = 0.087), - mean serum concentrations of fluoxetine and its metabolite (norfluoxetine) at baseline and 2 months later during combined antiviral treatment didn’t change significantly, - only the half-life of fluoxetine showed a significant reduction during combined antiviral therapy (*p* = 0.014)	National Center for Biotechnology Information (2012), Cohort study
Treatment with peginterferon-α-2a	methadone (CYP3A, 2C8 and 2D6)	24 with hepatitis C	- treatment did not alter the pharmacokinetic of methadone in patients, - increase exposure of total methadone by 10–15% was not statistically significant	Wu and Fleming (2011), Non-randomized
Treatment with peginterferon-α-2b	methadone (CYP3A, 2C8 and 2D6)	20 with hepatitis C	- a barely significant increase in total methadone exposure of 15–16% was observed after 4 weekly injection of peginterferon-α-2b - this increase was not clinically significant because there were no symptoms of methadone overdose	Ling et al. (2009), Non-randomized
Treatment with peginterferon-α-2a	theophylline (CYP1A2), tolbutamide (CYP2C9), mephenytoin (CYP2C19), debrisoquin (CYP2D6) and dapsone (CYP3A)	14	- theophylline AUC increased significantly but Cl/F difference was not significant, - no effect on the PK of any other probe drug	Schmitt et al. (2011), Cohort study
Treatment with INF-β	mephenytoin (CYP2C9 and 2C19 and induces 2C9, 2C19 and 3 A) and debrisoquin (CYP2D6)	10 with multiple sclerosis in the first stage	(S)/(R) mephenytoin ratio (*p* = 0.5) and debrisoquine MR (*p* = 0.4) were not statistically significant different before and during regular INF-β treatment	Zhuang et al. (2015), Non-randomized

**TABLE 14:** Impact of therapies with anti-TNF-α and -mabs on CYP substrates, explained totally or partially by modulation of CYP activity.

**Inflammation characterized by**	**Victim drugs (CYPs concerned)**	**Number of subjects**	**Potential effect of interaction**	**References and design**
Basiliximab	tacrolimus (CYP3A)	12 = treatment, 8 = control	- 63% increased tacrolimus trough concentration in basiliximab group at day 3 vs controls (*p* < 0.05), - tacrolimus through concentration decreased in basiliximab group 30 days after transplantation, - Authors suggest that basiliximab induced alteration in drug metabolism because its binding to IL-2R on activated T cells allows circulating IL-2 to bind to IL-2R on hepatic and intestinal cells resulting in a down-regulation of CYP3A4	Wen et al. (2020), Non-randomized
OKT3 (muromonab)	CyA (CYP3A)	17 = OKT3, 16 = controls	- on days 1 and 3, CyA through concentration did not differ but it was significantly higher in OKT3-group at day 5 as compared to control (*p* < 0.0001), - on days 7 and 10, CyA through level did not differ again	Tran et al. (2016), Case-control study
Adalimumab	duloxetine (CYP1A2 and 2D6)	22 years-old woman	- adalimumab was initiated for a refractory psoriasis but the peripheral neuropathy became unbearable leading to double the duloxetine’s dosage while she had a long-standing treatment by duloxetine and pregabalin, - authors did not suggest any interaction’s mechanism but it could be possible that the decrease of TNF-α by adalimumab led to a lift of the inhibition of CYPs, - no apparent interaction with pregabalin, which is eliminate by renal way	Lee et al. (2017), Case report
Infliximab	verapamil (CYP3A4, 1A2, 2C8, 2C9 and 2C18)	12 = RA with infliximab, 8 = RA controls, 12 = healthy controls	- serum CRP and IL-6 concentrations were significantly greater in RA patients who were on nonbiologic antirheumatic therapy compared with controls (*p* < 0.05 and *p* < 0.001, respectively), - CRP and IL-6 concentrations were not significantly different between RA patients taking infliximab and control subjects, - difference in RA patients who were on nonbiologic treatment in all PK parameters of verapamil, but it did not reach statistical significance but no difference between controls and RA patients who were taking infliximab, - infliximab did not show overall superiority to placebo on depressive symptom outcome	Davis et al. (2018), Case-control study
Infliximab	antidepressants	30 = infliximab, 30 = placebo		Wollmann et al. (2017), RCT
Secukinumab	midazolam (CYP3A)	24 = Psoriasis Area Severity Index (PASI) score >12 taking secukinumab	- secukinumab treat the immune-mediated disease by neutralizing the underlying inflammation and tissue destruction, - patients with PASI score >12 taking secukinumab, a decreased in IL-6 and CRP levels were observed after the start of treatment, - any change was seen in the PK parameters of midazolam before and after the administration of secukinumab, - PK parameters of midazolam in patients with psoriasis (study subjects) were close to those in found in healthy subjects in a previous study	Sifontis et al. (2002), Non-randomized
risankizumab	caffeine (CYP1A2), warfarin (CYP2C9), omeprazole (CYP2C19) and metoprolol (CYP2D6)	21	- risankizumab is an antibody that acts against IL-23 and it is involved in immune and inflammatory response thus, risankizumab inhibits its cells signalling pathway and the release of pro-inflammatory cytokines, - metabolic activity of CYP1A2, 2C9, 2C19, 2D6 and 3A4 were assessed before and 12 weeks after onset of treatment and any differences were observed, - authors conclude that treatment with risankizumab is not expected to cause CYP-mediated drug interactions	Vasquez and Pollak (1997), Non-randomized
tocilizumab	simvastatin (CYP3A)	12	- exposure to simvastatin was significantly reduced by approximately half at 1 and 5 weeks after tocilizumab infusion	Bruin et al. (2019), Randomized
sirukumab	midazolam (CYP3A), omeprazole (CYP2C19), warfarin (CYP2C9), caffeine (CYP1A2)	12	- administration of probe drugs 1 week before and 1, 3 and 6 weeks after sirukumab administration, - AUC of midazolam, omeprazole and S-warfarin decreased and those of caffeine increased as compared with those before sirukumab administration, - it was not because it is a CYP inducers, but because the inhibition by inflammation may be reversed by its IL-6 antagonism, - for CYP1A2, this result suggests that inflammation induce its metabolic activity, - authors suggest that, according to literature, IL-6 may have a biphasic impact on CYP1A2 activity depending on the IL-6 concentration, with an induction observed with low level of IL-6	Khatri et al. (2019), Non-randomized
dupilimumab	midazolam (CYP3A), omeprazole (CYP2C19), warfarin (CYP2C9), caffeine (CYP1A2) and metoprolol (CYP2D6)	13	- no impact of blockade of IL-4 and IL-13 signalling on the metabolic activity of CYP3A, 2C19, 2C9, 1A2 and 2D6	Mühlbacher et al. (2021), Non-randomized
biological disease-modifying antirheumatic drugs	4β-hydroxycholesterol (4βOHC) (CYP3A)	31 = TNF-α inhibitor, 5 = IL-6 inhibitor, 5 = B-cells inhibitors, 52 = controls	- levels did not change after the onset of any of the three treatments, - a trend was observed that lowest baseline 4βOHC levels (higher inhibition of CYP3A4 metabolic activity) showed highest relative increase in at follow-up and thus a highest regain in metabolic activity of CYP3A4 after initiation of treatment, - authors suggest that the absence of variation in 4βOHC levels in this study could be explained by the low level of inflammation in these patients because 4βOHC level in the study population at baseline was only 30% lower than in control groups	Girardin et al. (2012), Cohort study and case-control study
TNF-α inhibitor	4βOHC (CYP3A)	31	- CRP values were lower than before 3 months treatment, but the difference was not statistically significant (*p* > 0.2) and 4βOHC levels were not significantly affected (*p* > 0.9) by the initiation of treatment, - significant negative correlations were observed between 4βOHC and IL-1ra and IL-6 (*r* = -0.410, *p* = 0.022) and CXCL8 (*r* = −0.403, *p* = 0.025)	Chládek et al. (1999), Cohort study Same subject as in Girardin et al. (2012)
etanercept	CyA (CYP3A)	42-year-old male	-2.5-fold increase of clearance after initiation of etanercept	Yang et al. (2003), Case-report
daclizumab	caffeine (CYP1A2), warfarin (CYP2C9), omeprazole (CYP2C19), dextromethorphan (CYP2D6) and midazolam (CYP3A)	30 = multiple sclerosis	- daclizumab treatment had no effect on CYP1A2, 3C9, 2C19, 2D6 and 3 A activity in patients with multiple sclerosis as compared to before treatment	Hefner et al. (2015), Cohort study
sarilumab	Simvastatin (CYP3A)	19	- plasma exposure decreased by 45% in RA patients 1 week after sarilumab injection, as compared to baseline, - one dose led to decreased of CRP level and IL-6 inhibition and, thus, restauration of CYP3A enzyme activity	Harbrecht et al. (2005), Cohort study

#### Infection

Several studies have assessed the association between infection, represented by elevated levels of CRP, and PK variations of voriconazole. This is of particular interest and voriconazole therapeutic drug monitoring should thus be used to optimize clinical success and safety in these settings (Luong et al., 2016). Increased levels of CRP were correlated with increased voriconazole concentrations or decreased metabolic ratio of voriconazole/N-oxide and this could be explained by CYP2C19 and/or CYP3A downregulation, as voriconazole is mainly metabolized by these two CYPs (van Wanrooy et al., 2014; Encalada Ventura et al., 2015; Dote et al., 2016; Niioka et al., 2017; Vreugdenhil et al., 2018; Schulz et al., 2019). A positive correlation between inflammatory markers and voriconazole concentration was seen in adults, as well as with the severity of infection (van Wanrooy et al., 2014; Dote et al., 2016; Veringa et al., 2017; Gautier-Veyret et al., 2019). Drug metabolism appears to be influenced by the degree of inflammation and standardization of the classification of inflammatory markers elevation seems necessary (van Wanrooy et al., 2014; Niioka et al., 2017; Veringa et al., 2017; Gautier-Veyret et al., 2019). Indeed, voriconazole through concentration increased by 0.015 mg/L every 1 mg/L increase in CRP, and a recent meta-analysis showed that an increase in voriconazole through concentration of 6, 35 and 82% was associated with an increase in the CRP level of 10, 50 and 100 mg/L, respectively (van Wanrooy et al., 2014; Bolcato et al., 2021). As a final evidence to support of a correlation between inflammation and CYP downregulation, inflammation, and its resolution, decreased, and increased voriconazole clearance respectively, suggesting that the improvement of the inflammation allows a return to the baseline (Dote et al., 2016). However, no studies have investigated the duration of the resolution of inflammation-induced metabolic phenoconversion (Stanke-Labesque et al., 2020). This is an important limitation to allow individualization of treatment without therapeutic drug monitoring (TDM), as under-exposure to drug remains a risk (Stanke-Labesque et al., 2020).

CYP downregulation was also demonstrated as a consequence of sufficient inflammation and significant temperature elevation (Elin et al., 1975). Therefore, caution should be exercised in case of infection when administering CYP substrates, as this may result in toxicity and ADRs (Vozeh et al., 1978; Blumenkopf and Lockhart, 1983; Levine and Jones, 1983 1; Raaska et al., 2002; Haack et al., 2003; de Leon and Diaz, 2003; Jecel et al., 2005; Darling and Huthwaite, 2011; Espnes et al., 2012; Kwak et al., 2014; Leung et al., 2014; Takahashi et al., 2015; Clark et al., 2018; Khan and Khan, 2019).

Early works assessed the effect of an infection induced intentionally by lipopolysaccharides (LPS) injection on antipyrine pharmacokinetics, and several studies have assessed the impact of infection on psychotropic agents (clozapine, risperidone). The increase of clozapine levels, a CYP1A2 substrate, due to inflammation has been well studied and demonstrated (Raaska et al., 2002; Haack et al., 2003; de Leon and Diaz, 2003; Jecel et al., 2005; Pfuhlmann et al., 2009; Darling and Huthwaite, 2011; Espnes et al., 2012; Abou Farha et al., 2012; Leung et al., 2014; Kwak et al., 2014; Takahashi et al., 2015; ten Bokum et al., 2015; Hefner et al., 2016; Ruan et al., 2017; Clark et al., 2018; Ruan et al., 2018; Ruan et al., 2020). A positive and significant correlation between clozapine and CRP levels (*r* = 0.313, *p* < 0.01) was found, with a 2- to 6-fold increase in serum levels and the development of toxic symptoms, as well as improvement after dose reduction or infection recovery (Raaska et al., 2002; Haack et al., 2003; de Leon and Diaz, 2003; Jecel et al., 2005; Pfuhlmann et al., 2009; Darling and Huthwaite, 2011; Espnes et al., 2012; Kwak et al., 2014; Leung et al., 2014; Takahashi et al., 2015; ten Bokum et al., 2015; Hefner et al., 2016; Abou Farha et al., 2012; Ruan et al., 2017; Clark et al., 2018; Ruan et al., 2018; Ruan et al., 2020). Further investigations are needed concerning anticoagulant therapy, as only one case of severe bleeding in the context of infection was reported in the literature (Blumenkopf and Lockhart, 1983). First observation of a return to baseline metabolic activity after the end of the disruption that caused inflammation dates from 1985, with the gradual improvement of antipyrine clearance in days after the resolution of pneumonia (Sonne et al., 1985). Later, other authors demonstrated metabolic recovery after improvement of a liver fluke infection following praziquantel treatment (Satarug et al., 1996).

In hepatitis (Table 2B), a study suggested an overall downregulation of several hepatic CYPs and transporters with liver fibrosis progression, although the mechanisms of regulation differed and large inter-individual variation existed (Hanada et al., 2012). Indeed, this study assessed that the mRNA level was largely dependent on fibrosis stage and that the role of the different nuclear receptors tested is not the same in the hepatic expression of each CYP isoenzyme (Hanada et al., 2012). CYP3A4 downregulation during HCV infection has been well-described (McHorse et al., 1975; Tuncer et al., 2000; Latorre et al., 2002; Wolffenbüttel et al., 2004). Indeed, numerous studies have described a higher drug exposure of the two most commonly used immunosuppressants, tacrolimus and cyclosporine A, in patients with hepatitis and especially in those with viremia (Tuncer et al., 2000; Latorre et al., 2002; Wolffenbüttel et al., 2004). Moreover, when HCV is treated, CYP activities appear to return to baseline levels in several studies (McHorse et al., 1975; van den Berg et al., 2001; Kugelmas et al., 2003; Ueda et al., 2015; Kawaoka et al., 2016; Saab et al., 2016; Raschzok et al., 2016; Ueda and Uemoto, 2016; Smolders et al., 2017). Indeed, through concentration of tacrolimus decreased after initiation of HCV treatment, such as sofosbuvir, daclatasvir, asunaprevir, simeprivir, ribavirin and interferon, administered alone or in combination, and it required a dosage increase (Kawaoka et al., 2016; Raschzok et al., 2016; Saab et al., 2016; Smolders et al., 2017). Subgroups were identified, such as patients not responding to interferon with higher CYP3A downregulation related to higher levels of circulating cytokines, confirming that CYP modulation is proportional to intensity of inflammation (Morcos et al., 2013). However, conflicting results exist, and clinical recovery from acute liver disease was not accompanied by a corresponding recovery of drug-metabolizing capacity in a study (Breimer et al., 1975). This could be due to a lag between the return to baseline CYP levels and recovery, as clinical recovery from liver disease is not accompanied by a corresponding recovery of drug metabolizing capability (Breimer et al., 1975). Indeed, it is generally recognized that recovery half-lives are approximatively 20–50 h after mechanism-based inhibition and 40–60 h after enzyme induction (Imai et al., 2011).

Several studies have examined the impact of HIV on CYP metabolism (Table 2C) and have shown that several concomitant treatments and antiretroviral drugs metabolized by CYP3A have reduced metabolism in HIV-infected individuals, with an increased risk of ADRs. For instance, clindamycin clearance decreased from 0.27 in healthy volunteers to 0.21 L/h/kg in AIDS patients (*p* = 0.014) and a negative correlation between TNF-α and midazolam clearance was found (Gatti et al., 1993; Jones et al., 2010). Moreover CYP3A inhibitor (ketoconazole or ritonavir) and inducer (rifampicin) effects were less pronounced on antiviral PK in HIV-patients (Gatti et al., 1993; Grub et al., 2001; Jetter et al., 2010; European medicines agency; Packageinserts). It is important to characterize CYP3A modulation in HIV, as many antiviral treatments are metabolized by this pathway, and this could lead to efficacy or safety concerns. However, the AUC of atazanavir was lower in HIV-infected patients than in healthy volunteers and this could be explained by the absence of correlation between its oral clearance and inflammatory markers in a cohort study, the lack of identical study conditions (doses, sample schedule, meals … etc.) between the two groups and the fact that HIV infection was well-controlled (Packageinserts; Le Tiec et al., 2005; Venuto et al., 2018). Indeed, caffeine metabolism was not altered in HIV-infected patient compared with healthy volunteers, but was decreased in AIDS patients (Lee et al., 1993; Jones et al., 2010). Moreover, atazanavir was administered with the booster ritonavir to decrease its clearance, and the effect of inflammation could have been minimized.

More recently, some studies have shown increased plasma concentration of CYPs substrates (mostly CYP3A) during SARS-CoV-2 infection, which may have led to believe that there was a CYPs downregulation due to inflammation (Table 2D) (Cojutti et al., 2020; Cranshaw and Harikumar, 2020; Gregoire et al., 2020; Marzolini et al., 2020; Schoergenhofer et al., 2020; Testa et al., 2020). Indeed, the plasma concentrations of some CYP3A substrates (lopinavir, darunavir and direct oral anticoagulants) were significantly increased in patients with SARS-CoV-2 infection (Cojutti et al., 2020; Gregoire et al., 2020; Schoergenhofer et al., 2020; Testa et al., 2020). CRP and IL-6 were also associated with lopinavir concentrations and a trend toward a return to baseline was observed after treatment with tocilizumab (Marzolini et al., 2020; Schoergenhofer et al., 2020). Indeed, lopinavir through level in patients with SARS-CoV-2 infection was twice as high as in HIV patients but concentrations decreased when tocilizumab was administered (Marzolini et al., 2020; Schoergenhofer et al., 2020). However, the impact of inflammation induced by SARS-CoV-2 infection on lopinavir through concentration may be also due to increased orosomucoid levels (Boffito et al., 2021; Stanke-Labesque et al., 2021). Lopinavir is a highly protein-bound drug and the misinterpretation of its overexposure during inflammation could be explained by the fact that total and not unbound concentration was considered (Boffito et al., 2021; Stanke-Labesque et al., 2021). Furthermore, a case report described clozapine toxicity and increased clozapine level from 0.57 to 0.73 mg/L during SARS-CoV-2 infection (Cranshaw and Harikumar, 2020). However, no correlation was found between CRP and hydroxychloroquine plasma concentrations (Marzolini et al., 2020).

#### Vaccination

Regarding vaccination (Table 3), several reports and studies assessed variations of PK/PD parameters of drugs after vaccination, but data remain contradictory. Of the 31 articles included, 28 were exclusively about influenza vaccination while two were about concomitant vaccinations including influenza (pneumococcus, tetanus and hepatitis A). Only one article did not evaluate the influenza vaccination but reported on the impact of *tuberculosis* vaccination (BCG). No significant difference of CYP activity between before or after vaccination was shown in several studies (Britton and Ruben, 1982; Fischer et al., 1982; Goldstein et al., 1982; Patriarca et al., 1983; Stults and Hashisaki, 1983; Stults and Hashisaki, 1983; Hayney and Muller, 2003). In particular, the impact of vaccination on anticoagulants effects has been well-studied but the majority of studies showed no variation of PT time or INR (Farrow and Nicholson, 1984; Kramer et al., 1984; Gomolin, 1986; Raj et al., 1995; Poli et al., 2002; Paliani et al., 2003; Iorio et al., 2006; Jackson et al., 2007; MacCallum et al., 2007; Casajuana et al., 2008). However, the occurrence of bleeding events a few days after vaccination, when the PT time was previously stable, has been described (Kramer et al., 1984; Weibert et al., 1986; Carroll and Carroll, 2009). Moreover, the case of a patient hospitalized because of serum CPK level of 93,000 U/L during treatment with cerivastatin and bezafibrate or the occurrence of tramadol toxicity has been reported (Plotkin et al., 2000; Pellegrino et al., 2013). The patient had been vaccinated 5 days earlier (Plotkin et al., 2000). Other studies, few in number, have found an effect of vaccination on the PK of CYP substrates (Renton et al., 1980; Kramer and McClain, 1981; Gray et al., 1983). However, no study has correlated the data with pro-inflammatory markers.

#### Organs Diseases

The influence of liver and kidney function on disposition of drugs excreted by the liver and kidney is widely recognized and used to derive dosing adaptations. However, there is now an increasing appreciation that kidney impairment can also reduce non-renal clearance and alter the bioavailability of drugs predominantly metabolized by the liver (Nolin, 2008). Indeed, uremic toxin has been implicated in transcriptional, translational and acute posttranslational modifications of CYP, and it has been recognized that inflammation is a common feature in end-stage renal disease (ESRD) patients (Nolin, 2008; Stenvinkel and Alvestrand, 2002). For example, CYP3A activity increased post-dialysis, meaning that it is the presence of uremic toxin that is responsible for CYP downregulation and not the underlying disease (Nolin et al., 2006). An inverse relationship between hepatic CYP3A activity was found in this study, but it did not prove causality (Nolin et al., 2006). It indicates that uremia can be used as a surrogate for dialyzable toxins that contribute to alterations in CYP3A function (Nolin et al., 2006). Indeed, hemodialysis improved CYP3A activity with a 27% increase 2 h post-dialysis in uremic patients, suggesting that potential toxins responsible for this alteration were removed (Nolin et al., 2006). Authors suggested that this improvement occurred independently of transcriptional or translational modifications, contrary to what has been suggested previously (Nolin et al., 2006). However, as shown in Table 4, two studies found an association between the modification of CYP activity and inflammation in ESRD patients (Molanaei et al., 2012; Molanaei et al., 2018).

All studies in patients with liver disease described a decrease in CYP activity, compared to controls, as shown in Table 5. Indeed, several studies studied antipyrine, an old drug that is metabolized by multiple CYP (Branch et al., 1973; Farrell et al., 1979; Salmela et al., 1980; Teunissen et al., 1984; Schellens et al., 1989; Bauer et al., 1994; Grieco et al., 1998; Frye et al., 2006). They showed that CYP activity and antipyrine metabolism decreased only in severe disease compared to inactive cirrhosis, mild-moderate liver disease or healthy volunteers (Farrell et al., 1979; Bauer et al., 1994; Grieco et al., 1998). Moreover, chronic liver disease appeared to have a higher impact than an acute/reversible pathology (Branch et al., 1973). However, few studies have focused on a specific CYP substrate, and no studies found an association with inflammatory markers. One study demonstrated that CYP2C19, 2E1, 1A2 and 2D6 probe drugs concentrations were inversely correlated to the Child-Pugh score and another one demonstrated that phenacetin clearance decreased by 90% in patients with cirrhosis (Frye et al., 2006; Wang et al., 2010). Concerning CYP2C9, tolbutamide plasma levels increased by 10–20% and irbesartan AUC increased by 20–30% in cirrhotic patients (Ueda et al., 1963; Marino et al., 1998). The same results were found with CYP3A as diazepam clearance decreased in cirrhosis (Klotz et al., 1975). These variations may therefore be attributed to the loss of liver function due to tissue destruction. CYP metabolism appeared to be influenced by other organ’s disease, such as clozapine serum levels that increased by 2-fold during chronic obstructive pulmonary disease (COPD) exacerbation and antipyrine clearance that was significantly lower in patient with COPD and antitrypsin deficiency than in healthy volunteers (Laybourn et al., 1986; Leung et al., 2014). In addition, one study showed that inflammatory markers were inversely correlated with CYP1A2 and CYP2C19 activity but not with CYP2D6 and CYP2E1 activity in patients with congestive heart failure (Frye et al., 2002).

Some studies conducted in critically ill patients (Table 8), showed that CYP1A2 and 3A metabolic activity were downregulated, and that it may be proportional to the severity and reversibility of the illness (Shelly et al., 1987; Toft et al., 1991; Kruger et al., 2009). For instance, theophylline clearance decreased by 10–66%, atorvastatin AUC increased by 15-fold, and clopidogrel active metabolite decreased by 48-fold, raising concerns about treatment efficacy (Toft et al., 1991; Kruger et al., 2009; Schoergenhofer et al., 2018). However, a systematic review reported that 20–65% of critically patients had an increased renal clearance, defined as a creatinine clearance greater than 130 ml/min/1.73 m^2^ (Bilbao-Meseguer et al., 2018). This underscores the fact that inflammation has a different effect on drug clearance through the different mechanisms of drug elimination.

#### Diabetes

In diabetes (Table 9), CYP metabolism has been shown to be downregulated (Salmela et al., 1980; Pirttiaho et al., 1984). Indeed, antipyrine metabolism was decreased compared with controls in several studies (Salmela et al., 1980; Pirttiaho et al., 1984; Zysset and Wietholtz, 1988). One study using a cocktail approach showed that CYP2B6, CYP2C19 and CYP3A activity decreased, CYP1A2 and CYP2C9 activity increased, and CYP2D6 and CYP2E1 activity was unaffected in type II diabetes (T2D) (Gravel et al., 2019). However, conflicting results exist with tolbutamide and paracetamol half-lifes which were unchanged and increased respectively (Ueda et al., 1963; Adithan et al., 1988). Regarding CYP3A, one study found no impact on amlodipine or immunosuppressant metabolism while nisoldipine clearance was decreased (Wadhawan et al., 2000; Preston et al., 2001; Marques et al., 2002; Akhlaghi et al., 2012). The underlying mechanisms are associated with systemic inflammation and inflammatory cytokines. Indeed, it is well-established that chronic inflammation is involved in the pathophysiology of diabetes and the more complex condition of metabolic syndrome (Gravel et al., 2019). TNF-α can lead to the development of diabetes by affecting insulin action, and levels of inflammatory cytokines and markers are reported to be increased in diabetes patients (Darakjian et al., 2021). In a multivariate analysis, IFN-γ, IL-1β, IL-6 and TNF-α were associated with CYP activities, depending on the CYP isoenzyme (Gravel et al., 2019). However, type I (T1D) and type II diabetes did not appear to have the same impact on CYP metabolism (Dyer et al., 1994; Korrapati et al., 1995; Lucas et al., 1998; Zysset and Wietholtz, 1988; Matzke et al., 2000; Sotaniemi et al., 2002; Wang et al., 2003). The impact of inflammation may be different partly because of obesity, which is more common in T2D (Wang et al., 2003). Indeed, obese patients had a 40% increase in CYP2E1 activity (Lucas et al., 1998; Wang et al., 2003). CYP2E1 increased activity could also be attributed to hypo-insulinemia, as administration of insulin reverses this induction at the mRNA level (Lucas et al., 1998). Moreover, moderate controlled T1D had comparable CYP2E1 activity to healthy volunteers (Wang et al., 2003). This was confirmed in other studies that showed an unaffected metabolic clearance rate of antipyrine in well-controlled (by insulin) T1D (Zysset and Wietholtz, 1988; Sotaniemi et al., 2002). This could also be explained by insulin supplementation and the subsequent correction of ketones that leads to a return to baseline level for CYP2E1 expression (Wang et al., 2003). Indeed, ketones have been shown to be an important modulator of CYP2E1 by enhancing its protein expression and mRNA level (Wang et al., 2003). This has been confirmed with CYP1A2, where fluctuations in growth hormone levels, hyperketonemia and variation in glucose metabolic steady state and HbA1C levels may contribute to these changes (Bechtel et al., 1988; Korrapati et al., 1995; Matzke et al., 2000). The difference in classification criteria for T1D and type 2 diabetes may explain the inconsistent findings (Matzke et al., 2000). Further studies to discriminate between these two entities are needed (Zysset and Wietholtz, 1988).

Overall, CYP3A, 2C19 and 2B6 activity appear to be downregulated while CYP1A2 activity was increased and CYP2D6 activity was unchanged in diabetic patients (Bechtel et al., 1988; Urry et al., 2016; Gravel et al., 2019). Conflicting results remain regarding CYP2C9 and CYP2E1 (Ueda et al., 1963; Adithan et al., 1988; Lucas et al., 1998; Gravel et al., 2019).

#### Auto-Immune Diseases

Few studies observed the impact of auto-immune disease on CYP activities, such as psoriasis, systemic lupus erythematosus (SLE), Behçet’s disease, rheumatoid arthritis (RA), Crohn’s disease and celiac disease (Table 10). In contrast to what has been observed for CYP2D6 in other inflammatory states, two studies observed CYP2D6 downregulation in patient with SLE (Idle et al., 1978; Baer et al., 1986). However, these studies have some limitations, such as the presence of concomitant medications inhibiting the metabolism of CYP2D6 and the absence of adequate randomization (Baer et al., 1986). Even though RA is one of the most prevalent chronic inflammatory disease, only two case-control studies were found in the literature studying the impact of RA on the PK and PD of verapamil and losartan, respectively (Mayo et al., 2000; Daneshtalab et al., 2006; Smolen et al., 2016). Verapamil is metabolized by CYP3A and 1A2 into norverapamil (Tracy et al., 1999). Verapamil and norverapamil metabolism has been shown to be reduced in patients with RA compared to healthy volunteers (Mayo et al., 2000). Verapamil was not more dromotropic or hypotensive in RA patients (Mayo et al., 2000). Inhibition of CYP2C9 was proportional to RA disease severity in another study, but this was not accompanied by reduced clinical response after losartan administration (Daneshtalab et al., 2006). Same results were found in patients with Behcet’s disease. Indeed, one study observed downregulation of CYP2C9 in Behcet’s patients (Goktaş et al., 2015). However, losartan’s MR in nine patients with Behçet’s disease taking colchicine were similar to those not taking colchicine (Goktaş et al., 2015). This may be because the drug had been taken for only 2 weeks (Goktaş et al., 2015).

In Crohn’s disease, S-verapamil concentration was higher than R-verapamil while the opposite was found in normal conditions and higher plasma levels of propranolol were found in Crohn’s with reduced metabolic activities of CYP1A2, 2D6 and 2C19 (Schneider et al., 1976; Sanaee et al., 2011). Furthermore, there were no difference between healthy controls and Crohn’s disease patients in remission, implying that CYP downregulation is proportional to disease severity and that recovery resulted in a return to baseline metabolic activity (Sanaee et al., 2011). Norverapamil goes through the same process and it is expected that the enantiomers ratio of norverapamil to verapamil remains unchanged (Sanaee et al., 2011).

Celiac disease is an autoimmune disease that is triggered by an immune response to gluten and may result in increased morbidity or mortality (Lebwohl et al., 2018). The reduction in intestinal CYP3A content during celiac disease and its increase after a gluten-free diet indicate that local inflammation reduced CYP3A activity but that it returns to baseline with disease improvement (Lang et al., 1996).

#### Surgery

The impact of surgery on concomitant treatment and analgesia management has been assessed in several studies (Table 11). Surgery is associated with an inflammatory response due to muscle or tissue injury to induce repair, regeneration and growth and so inflammatory markers increase after surgery, but not equally (Tidball, 2005; Stavropoulou et al., 2018). IL-1β was only detected during the early perioperative period and for a very short time (Baigrie et al., 1992). IL-6 plasma level peaked 4–48 h after surgery and declined drastically by 48–72 h in all patients without any postoperative complication (Baigrie et al., 1992). CRP level rose more slowly postoperatively compared with the cytokine levels (IL-6, TNF-α and IL-1β) (Bergin et al., 2011). Acute inflammation after elective surgery was associated with a significant decrease in CYP3A metabolic activity (Haas et al., 2003). A recent study with a cocktail approach has concluded that there is an isoform specific impact of inflammation on CYP activities (Lenoir et al., 2020). Indeed, this study showed that CYP1A2, CYP2C19 and CYP3A activities decreased significantly by 53, 57 and 61%, whereas CYP2B6 and CYP2C9 activities increased significantly by 120 and 79% (Lenoir et al., 2020). However, surgery did not significantly impact CYP2D6 activity (Lenoir et al., 2020). These findings were confirmed by a case report that showed a toxic increase in clozapine levels 4 days after surgery and by authors who further showed that clopidogrel efficacy was reduced in patients undergoing percutaneous coronary intervention, because clopidogrel must be bioactivated by CYP2C19 to be effective (Bernlochner et al., 2010; Leung et al., 2014; Mostowik et al., 2015).

#### Cancer

Inflammation is linked to all stages of cancer (risk of development, initiation, invasion, metastasis and mortality) as highlighted in Table 12 (Harvey and Morgan, 2014). Certain immune-mediated diseases have been associated with cancer such as inflammatory bowel disease (IBD), chronic infection by Helicobacter pylori and chronic psoriasis associated with an increased risk of colorectal, gastric and skin cancer, respectively (Harvey and Morgan, 2014). The first pro-cancer immune signals are via tumor cells that successively produce cytokines and act to increase transcription factors, induce epigenetic changes and initiate angiogenesis (Harvey and Morgan, 2014). Cytokines are involved from neoplastic transformation of cells to tumor progression and metastasis, and are thus involved in several cellular events leading to cancer (Kacevska et al., 2008). These signals and others induced to respond to cancer are opposed by antigen-presentating cell-mediated anticancer immune responses (Harvey and Morgan, 2014). Moreover, the greater the antitumoral response is, the more the cancer outcome is improved whereas some T-cells subsets are associated with tumor promotion (Harvey and Morgan, 2014). Some cytokines have tumor-promoting, antitumor effects or both (Kacevska et al., 2008). Some cytokines could be produced by the tumor itself (Kacevska et al., 2008). Inflammation has therefore a pivotal role in cancer and the proliferation of malignant cells by a dynamic equilibrium in the tumor environment (Harvey and Morgan, 2014). Cytokines present in the tumor environment are also launched in the systemic circulation and have general effects on the function of distant organs such as the liver (Kacevska et al., 2008). Inflammatory markers levels are dependent on tumor types, but high level of CRP, IL-6, IL-1β have been associated with poor prognosis (Kacevska et al., 2008). Some results suggest that high IL-6 is associated with decreased CYP3A metabolic activity but can also nonspecifically downregulate CYP-dependent drug metabolism (Chen et al., 1994). CRP and α-glycoprotein were also negatively correlated with CYP3A activity and cancer patients with significant acute-phase response may have reduced CYP3A drug metabolism, which may have implications for the safety and efficacy of chemotherapy (Rivory et al., 2002; Charles et al., 2006; Alexandre et al., 2007). Inflammatory status and lymphocyte count should thus be included in the evaluation of the benefit/risk ratio before the initiation of a cytotoxic chemotherapy (Alexandre et al., 2007). Concerning CYP2C19, studies showed that CYP2C19 activity was not solely predicted by the genotype in cancer patients (Williams et al., 2000; Helsby et al., 2008; Burns et al., 2014). Indeed, CYP2C19 activity was reduced in cancer patients, with a discordance between the measured phenotype and the predicted phenotype from the genotype. However, no significant correlation was found between CYP2C19 activity and the levels of cytokine, whereas this was the case for voriconazole through concentration (Helsby et al., 2008; Burns et al., 2014; Yasu et al., 2017; Mafuru et al., 2019). The mechanism behind the decrease of CYP2C19 activity observed in cancer patients may be related to the inflammatory response even though it remains debated (Helsby et al., 2008; Burns et al., 2014; Yasu et al., 2017; Mafuru et al., 2019). Other authors showed that cancer has no impact on CYP1A2 metabolic activity as compared to liver disease or infection (Wang et al., 2010).

#### Therapies With Immunomodulator, anti-TNF-α and -Mabs

As biological therapies aim to decrease the underlying inflammation of the disease, interleukins (IL) injections are expected to have an impact on CYP activity, as underlined in Table 13. As an example, IL-2 doses of 9–12 × 10^6^ units daily may downregulate CYP activities in patients with HIV infection and cancer in whom this treatment is administered to boost the immune system (Piscitelli et al., 1998; Elkahwaji et al., 1999). Conflicting results exist regarding IFN administration, with a discrepancy between acute and chronic treatment (Williams and Farrell, 1986; Williams et al., 1987; Jonkman et al., 1989; Israel et al., 1993; Hellman et al., 2003; Sulkowski et al., 2005; Gupta et al., 2007; Furlanut et al., 2010; Brennan et al., 2013). However, case reports and more specific studies assessing CYP metabolic activity lean toward CYP downregulation and care must be taken to avoid interactions and ADRs (Craig et al., 1993; Adachi et al., 1995; Serratrice et al., 1998; Hassan et al., 1999; Becquemont et al., 2002). The level of anticoagulation should be closely monitored when interferon is given together with warfarin, as it appears that CYP are downregulated (Adachi et al., 1995; Serratrice et al., 1998). Additionally, the timing of IFN-α administration relative to concomitant chemotherapy should be considered to avoid a decrease in CYP3A4 and 2B6 activity and thus to achieve better efficacy (Hassan et al., 1999). For example, interferon-α-2b inhibits CYP1A2, 2D6 and 2C19 and these findings pose new challenges for patients on these therapies with respect to PK interaction with concomitant drugs commonly used (Islam et al., 2002). Further studies are needed to measure the impact of IFN and new cytokine therapies coming on the market on CYP activities. Cytokines act on CYP in an isoform-specific manner, and it is likely that IFN or IL modulate different CYP while they have no impact on others. Moreover, it is crucial to understand whether the modulation of CYP activity is due to this kind of therapy, to the underlying disease which may be inflammatory, or to its resolution by these same therapies (reduction of inflammation caused by the disease).

The impact of–mabs therapies are summarized in Table 14. Monoclonal antibodies have a high degree of specificity against an antigen or an epitope (National Center for Biotechnology Information, 2012). In 2018, more than sixty therapeutic monoclonal antibodies were approved and used in the United States for their action against specific immune cells such as lymphocytes and cytokines or against specific enzymes, cell surface transporters or signaling molecules (National Center for Biotechnology Information, 2012). Consequently, a number of studies have examined the impact of monoclonal antibodies on CYP metabolic activity, assuming that these drugs, by reducing inflammation, return CYP metabolic activity to baseline (Ling et al., 2009; Schmitt et al., 2011; Wu and Fleming, 2011; Zhuang et al., 2015; Tran et al., 2016; Lee et al., 2017; Wen et al., 2020) (Table 14).

A return to baseline level after treatment of inflammation was not always observed (Wollmann et al., 2017; Davis et al., 2018). A lag was observed in some cases, such as basiliximab coadministration, which increased tacrolimus through concentration on day 3 but decreased on day 30 (Sifontis et al., 2002). Moreover, OKT3 (also known as muromonab, a CD3 receptor antibody) treatment transiently increased CyA through concentration, and authors suggested that OKT3 inhibits CYP3A4 metabolic activity by inducing transient cytokine release (Vasquez and Pollak, 1997). No changes were observed in drugs PK parameters before and after monoclonal antibodies administration, possibly because CYP metabolic activity was similar in psoriasis disease and in healthy volunteers (Bruin et al., 2019; Khatri et al., 2019). However, these therapies are used for a variety of diseases, with different levels of proinflammatory markers. In addition, a recently published study assessed the impact of clazakizumab, an anti-IL-6 antibody, in kidney transplant recipients with antibody-mediated rejection (ABMR) on CYP3A and CYP2C19 activity by pantoprazole and on tacrolimus and CyA concentrations (Mühlbacher et al., 2021). In contrast to earlier observations, prolonged blockade of IL-6 did not enhance CYP metabolism (Mühlbacher et al., 2021). This could be because the included patients did not have systemic inflammation before initiation of clazakizumab, with IL-6 and CRP levels in the normal range (Mühlbacher et al., 2021). Thus, clazakizumab did not increase CYP metabolism because the included patients had unaltered CYP expression, as ABMR may be different from other disease states, such as infection or autoimmune disease, where systemic inflammation is present (Mühlbacher et al., 2021).

## Discussion and Perspectives

Our systematic review identified 218 publications that evaluated the impact of inflammation on CYP activities which we divided into 17 sources of inflammation. Indeed, current literature suggests that cytokine signalling pathways differ according to the trigger of inflammation, leading to heterogeneous effects on CYP activity, with different magnitude, potency and time-course (de Jong et al., 2020; Stanke-Labesque et al., 2020). This analysis allowed us to identify areas where the literature is abundant, such as infections like pulmonary infection, hepatitis or HIV and for some therapeutic agents like immunosuppressants or clozapine, and others where further research is needed, such as for auto-immune diseases, and other specific diseases such as diabetes or the anti-inflammation treatments.

Our analysis also identified that studies should be more specifically conducted to assess whether resolution of inflammatory episodes allows a return to baseline of CYP activities. Indeed, inflammatory diseases are chronic, but with a possibility of remission, and acute inflammatory events can punctuate life (infection, surgery, cancer…). A better understanding of the mechanisms of modulation and return to the initial state would make it possible to anticipate changes in the PK of concomitant treatments at different phases of the disease or of the patient’s life. This could be done through the impact of anti-inflammatory treatments as well as monoclonal antibody therapies. These therapies are relatively new and much remains to be discovered, but they are highly targeted, and the impact of these different molecules could be isoform specific.

Our literature review highlighted the different effect of inflammation according to the CYP considered. Several studies have investigated the impact of infection on drugs of the nervous systems, mainly CYP2D6 substrates without always showing a significant impact. It now appears that CYP2D6 activity is not modulated by inflammation and this is confirmed in chronic hepatitis C patients where downregulation is linked to the presence of liver kidney microsomal type 1 (LKM-1) antibodies (Girardin et al., 2012). LKM-1 antibodies are often produced during chronic HCV infection and appear to be proportional to liver disease severity (Girardin et al., 2012). Moreover, it is well-known that CYP2D6 has an important inter- and intra-individual variability, in accordance with the available literature (Chládek et al., 1999). All sources of inflammation combined, the most studied CYP was CYP3A, which is in fact the CYP that metabolizes nearly 50% of the drugs on the market. Patients with inflammation/infection are, however, prone to receiving multiple drugs, and the impact on other CYPs should be carefully evaluated, in particular in critically ill patients or patients at different stages of HIV, where data is scarce. Studies should also be careful to exclude the impact of co-medications (CYP inhibitor and inducer) as a confounding factor.

In organ diseases, current studies in liver diseases have not been able to determine whether CYP downregulation is caused by a decrease of CYP content or not, and in renal diseases it was not possible to identify whether the modulation of CYP activity was rather due to elimination issues (Farrell et al., 1979; Yang et al., 2003). Therefore, it is challenging to study inflammation as an independent factor in PK variability and not as a consequences of organ damage.

Our literature review also found that inflammation is a complex process, which is expressed differently depending on the disease and conditions and therefore, extrapolation between different types of inflammation should be avoided. Indeed, the hepatic expression of CYP2C19 could for example be regulated by other tumor-associated inflammatory factors than those regulating CYP3A (Burns et al., 2014). Moreover, different levels of inflammation led to different magnitudes of voriconazole through concentration increases for instance in association with CRP levels (van Wanrooy et al., 2014; Bolcato et al., 2021). In most studies, significant changes in CYP activities occurred in the presence of severe inflammation, characterized by elevated levels of inflammatory markers or a severe disease state, such as AIDS, advanced cancer or polytrauma patients (Gatti et al., 1993; Lee et al., 1993; Farrell et al., 1979; Grieco et al., 1998; Bauer et al., 1994; Harbrecht et al., 2005; Charles et al., 2006; Alexandre et al., 2007; Helsby et al., 2008; Abou Farha et al., 2012; ten Bokum et al., 2015; Hefner et al., 2015; Yasu et al., 2017; Gautier-Veyret et al., 2019). A minority of studies have evaluated the impact of inflammation on drugs PK and metabolism as an independent factor of variability, as only a few have included inflammation factors as covariates, such as biomarkers of renal or liver function (Stanke-Labesque et al., 2020).

Additionally, inflammation may have a different impact on CYPs activities depending on their baseline activity and on genotypic and environmental factors, such has concomitant treatments. Indeed, inflammation further increased the perampanel concentration/dose (C/D) ratio in patients not treated with drug inducers (Yamamoto et al., 2018). Voriconazole is also metabolized by highly polymorphic CYPs and inflammatory marker levels have a differential impact on voriconazole trough concentration whether patients are extensive, intermediate or ultra-rapid metabolized for CYP2C19 (Veringa et al., 2017). Moreover, a recent meta-analysis showed that voriconazole trough concentrations were independently influenced by both CYP2C19 and CYP3A4 genotype, considered individually or by a combined genetic score, in addition to CRP levels (Bolcato et al., 2021). In contrast, another cohort study showed that voriconazole overdoses were significantly associated with elevated CRP levels (>96 mg/L) but that CYP2C19 and CYP3A4 genotype, considered alone or combined in a genetic score, were not significantly different between overdose and non-overdose patients (Gautier-Veyret et al., 2019). Therefore, inflammation and pharmacogenomics may mutually minimize their reciprocal influence on CYP phenotype. Indeed, genotype did not predict correctly the phenotype in patients with inflammatory disease and the effect of inflammation was not as important as expected in CYP variants carriers (Helsby et al., 2008; Goktaş et al., 2015; Burns et al., 2014; O’Neil et al., 2000; Williams et al., 2000; ). Consequently, inflammation could induce dynamic phenoconversion, characterized by dynamic phenotype-genotype mismatch, and studies examining the impact of inflammation on CYPs should assess CYP genotypes and phenotypes as covariates. It should however be pointed out that most of the included studies did not take into account routine treatment given to treat the diseases themselves.

Predictive models based on known interactions between molecular, environmental and lifestyle data by computational algorithm are increasingly developed to support the decision to individualize treatment (Iriart, 2019). Simulation of the concentration-time profiles of a drug and its metabolite(s) and concomitant estimation of PK parameters using dynamic physiologically based pharmacokinetic (PBPK) models allow prediction of plasma concentration curves (Sager et al., 2015). There are increasing developments in regulatory guidances (Sager et al., 2015). Inflammatory disease is an example of a special population and numerous PBPK models have been developed and validated to predict IL-6 mediated drug-disease (Machavaram et al., 2013; Xu et al., 2015; Jiang et al., 2016; Radke et al., 2017; Xu et al., 2018; Machavaram et al., 2019). While IL-6 appears to be the key element in modulating CYP activities during inflammation, a recent study developed a model that predicted the impact of systemic CRP levels on CYP3A4 and CYP2C19 activities (Simon et al., 2021). Optimal drug use leads to takes into account the contribution of covariates to predict the dose needed to achieve a target concentration and thus reduce the inter- and intra-individual variability in drug response (Darwich et al., 2021).

This review focuses on CYP regulation, but other mechanisms, such as enzymes and transporters, involved in drug absorption, distribution, metabolism and elimination may be involved in changes in drugs PK during inflammatory states, although they are less studied. Studies described changes in plasma protein binding and renal excretion during inflammation that could affect CYP substrates metabolism (Gorski et al., 2000; Hefner et al., 2015; Helland et al., 2018). Plasma protein binding may influence total clearance for low-extraction drugs but not unbound clearance and may or may not influence half-life, depending on clearance and volume of distribution (Boffito et al., 2021). The unbound concentration and not the total concentration must be considered when assessing drug exposure to a highly protein-bound drug, otherwise there is a risk of misinterpretation of lopinavir overexposure (Boffito et al., 2021; Stanke-Labesque et al., 2021). For example, by taking into account plasma protein concentration, the authors concluded that CyA biotransformation by CYP3A may be downregulated by diabetes (Akhlaghi et al., 2012). Decreased albumin concentration may increase the unbound concentration in diabetics, which should theoretically increase CyA metabolic clearance (Akhlaghi et al., 2012). But the lower production of almost all metabolites has shown that the correct hypothesis is rather a reduced CYP activity (Akhlaghi et al., 2012). In fact, CyA metabolites that involved amino acid 1 showed significantly lower dose-normalized AUC values in diabetic patients compared with nondiabetics suggesting that CYP3A4 metabolic activity was not decreased (Mendonza et al., 2008). Its dose-adjusted metabolite-parent concentration ratio was decreased in the diabetic groups, but no difference was found concerning doses and trough levels of CyA in a retrospective study (Wadhawan et al., 2000; Akhlaghi et al., 2012).

Phase 2 drug metabolic enzymes appear to be affected in a cytokine-specific manner, as infection resulted in a significant downregulation of several genes encoding hepatic uridine 5′-diphospho-glucuronosyltransferases (UGT) (Stanke-Labesque et al., 2020). Pregnane X receptor (PXR) and constitutive androstane receptor (CAR), two nuclear receptors, are also cytokine dependent and mediate the expression of glutathione S-transferases (GST), UGTs and sulfo-transferases (SULT) in humans (Wu and Lin, 2019). However, unlike voriconazole, posaconazole’s PK did not appear to be influenced by inflammation. This could be explained by a metabolism by phase 2 enzymes mainly (Märtson et al., 2019). Literature reviews on physiological changes related to drug PK and PD during inflammation may be useful to determine what investigations are needed to complement the data in the literature, such as the impact of inflammation on P-gp and other drug transporters, as one study showed that an increase in bioavailability due to downregulation of P-gp could not be ruled out (Sanaee et al., 2011).

Moreover, hepatic transporters that belong to ATP-binding cassette (ABC) and solute carrier (SLC) transporters have been shown to be significantly reduced during inflammatory states in animal and in-vitro studies (Stanke-Labesque et al., 2020). For instance, animals studies have shown that mRNA levels of MRP, OATP or BSEP were decreased in mice during inflammation (Wu and Lin, 2019). NF-κB, a transcription factors involved in the mechanism of action of cytokines on metabolizing enzyme gene expression, is also known to regulate the expression of numerous ABC and SLC transporters, including ABCB1 in humans and MDR1, MRP, BCRP, OATP, NTCP in rats and mice (Wu and Lin, 2019).

Given all of the above, it should be acknowledged that our literature search has some limitations. First, the completeness of the search cannot be guaranteed as we only searched one database and only published articles. Second, there is inevitably heterogeneity between the studies selected due to the different methodologies employed and low comparability between the studies identified. In addition, the diversity of the sources of inflammation studied and assessment of the clinical impact severity limits the robustness and generalizability of the results. Interpretations should therefore be addressed with particular caution.

## Conclusion

This systematic literature review shows that inflammation is a major contributing factor to CYP metabolic activity variations. The proportion of the drug cleared by CYP metabolism, the patient’s genotype and concomitant medications should also be taken into account.

Compelling evidence suggests that inflammation has a differential impact on the various CYP isoforms with a different magnitude. CYP3A and CYP2C19 are downregulated and inflammation has no impact on CYP2D6 activity. Regarding other main CYPs, the impact remains unclear and requires further investigation. Moreover, the effect of inflammation depends on its severity and the inflammatory markers released, even if this remains debated. Indeed, the origin of the inflammation may differ as well as the inflammatory mediators involved, possibly leading to different impact on CYP activities. The reason why some CYP metabolic activities were modulated in some diseases and not in others may be partly explained by this heterogeneity in inflammatory markers.

Nonetheless, some results are still debated such as the impact of vaccination and infection, and further investigations are required to well characterize the impact of inflammation on CYP activity.

CYP is a major source of interindividual variability, and it appears crucial to be able to predict their activity to individualize drug dosing and take into account the patient’s underlying pathophysiological conditions and the PK characteristics of the drug concerned. Measurement of inflammation induced CYP phenoconversion and the development of endogenous markers of CYP metabolism should enable the measurement of CYP activity variation due to disease progression and could have implications for personalized medicine and provide new opportunities.

To conclude, inflammatory conditions in patients are a major factor to be considered to predict variability in drug response and avoid efficacy or safety issue in clinical practice.

## Data Availability

The original contributions presented in the study are included in the article/Supplementary Material, further inquiries can be directed to the corresponding author.
